# BI 456906: Discovery and preclinical pharmacology of a novel GCGR/GLP-1R dual agonist with robust anti-obesity efficacy

**DOI:** 10.1016/j.molmet.2022.101633

**Published:** 2022-11-07

**Authors:** Tina Zimmermann, Leo Thomas, Tamara Baader-Pagler, Peter Haebel, Eric Simon, Wolfgang Reindl, Besnik Bajrami, Wolfgang Rist, Ingo Uphues, Daniel J. Drucker, Holger Klein, Rakesh Santhanam, Dieter Hamprecht, Heike Neubauer, Robert Augustin

**Affiliations:** 1 Boehringer Ingelheim Pharma GmbH & Co. KG, Birkendorfer Str. 65, 88400 Biberach an der Riβ, Germany; 2 Lunenfeld-Tanenbaum Research Institute, University of Toronto, Mount Sinai Hospital, 600 University Avenue, Toronto, Ontario M5G 1X5, Canada; 3 Boehringer Ingelheim Research Italia, Via Lorenzini 8, 20139 Milano, Italy

**Keywords:** Glucagon, Glucagon-like peptide-1, Obesity, G protein coupled receptor, Peptide

## Abstract

**Objective:**

Obesity and its associated comorbidities represent a global health challenge with a need for well-tolerated, effective, and mechanistically diverse pharmaceutical interventions. Oxyntomodulin is a gut peptide that activates the glucagon receptor (GCGR) and glucagon-like peptide-1 receptor (GLP-1R) and reduces bodyweight by increasing energy expenditure and reducing energy intake in humans. Here we describe the pharmacological profile of the novel glucagon receptor (GCGR)/GLP-1 receptor (GLP-1R) dual agonist BI 456906.

**Methods:**

BI 456906 was characterized using cell-based *in vitro* assays to determine functional agonism. *In vivo* pharmacological studies were performed using acute and subchronic dosing regimens to demonstrate target engagement for the GCGR and GLP-1R, and weight lowering efficacy.

**Results:**

BI 456906 is a potent, acylated peptide containing a C18 fatty acid as a half-life extending principle to support once-weekly dosing in humans. Pharmacological doses of BI 456906 provided greater bodyweight reductions in mice compared with maximally effective doses of the GLP-1R agonist semaglutide. BI 456906’s superior efficacy is the consequence of increased energy expenditure and reduced food intake. Engagement of both receptors *in vivo* was demonstrated via glucose tolerance, food intake, and gastric emptying tests for the GLP-1R, and liver nicotinamide N-methyltransferase mRNA expression and circulating biomarkers (amino acids, fibroblast growth factor-21) for the GCGR. The dual activity of BI 456906 at the GLP-1R and GCGR was supported using GLP-1R knockout and transgenic reporter mice, and an *ex vivo* bioactivity assay.

**Conclusions:**

BI 456906 is a potent GCGR/GLP-1R dual agonist with robust anti-obesity efficacy achieved by increasing energy expenditure and decreasing food intake.

## Highlights

• BI 456906 is a novel, acylated GCGR/GLP-1R dual agonist containing a C18 fatty acid.
• BI 456906 doses produced greater bodyweight reductions compared with semaglutide.
• Increased energy expenditure and decreased food intake drove BI 456906-induced weight loss.
• GLP-1R engagement was shown through glucose tolerance, food intake and gastric emptying.
• Liver *Nnmt* expression and plasma amino acids and FGF-21 demonstrated GCGR engagement.

## 1 Introduction

One of the greatest healthcare challenges of our time is the obesity epidemic, with an estimated prevalence of approximately 40% in the adult US population [1] and 25% in the adult European population [2], with an associated decrease in life-expectancy [3]. Obesity promotes the emergence of associated complications, such as type 2 diabetes, cardiovascular diseases, and non-alcoholic liver steatohepatitis (NASH), as well as certain cancers, imposing a considerable burden on healthcare systems [[3], [4], [5], [6], [7]]. Despite major advances in the treatment of cardiometabolic diseases, cardiovascular disease and type 2 diabetes still represent two of the main causes of death in Western societies [8], indicative of the unmet need for efficacious treatment. With the recognition of obesity as a chronic, relapsing, progressive disease [9], therapeutic interventions are urgently needed.

Despite major efforts, pharmacotherapies for obesity have historically faced continued challenges associated with limited efficacy and, more importantly, clinically relevant safety liabilities. The development of pharmacological mimetics of gastrointestinal peptide hormones involved in energy homeostasis [[10], [11], [12]] has paved the way to overcoming the historical failures associated with obesity pharmacotherapies [10]. Among these, endocrine peptides derived from preproglucagon are an important subgroup, including glucagon-like peptide-1 (GLP-1), glucagon, and oxyntomodulin. The discovery that GLP-1 receptor agonists (GLP-1RAs), previously approved for the treatment of type 2 diabetes [14,15], reduce energy intake alongside increasing insulin secretion and inhibiting gastric emptying [13], reinvigorated the field of obesity pharmacotherapy, with the development and approval of the long-acting GLP-1RAs liraglutide and semaglutide [16,17].

Although the class of GLP-1RAs and other clinically investigated peptide hormone mimetics (e.g. analogs of PYY or amylin) reduce energy intake, long-term weight loss and maintenance in people with obesity may require strategies that also promote preservation of or increased energy expenditure [18,19]. Obesity is considered to be a consequence of a positive energy balance as a result of a continued adaptation of both energy intake and expenditure, that favors weight gain or re-gain after weight loss [18,20]. The concept of simultaneously targeting both energy expenditure and intake to drive weight loss has been demonstrated with the peptide hormone oxyntomodulin, an endogenous, weak GLP-1R and glucagon receptor (GCGR) dual agonist. Derived from proglucagon, oxyntomodulin is a naturally occurring 37 amino acid peptide, containing 29 amino acids from the glucagon sequence with an 8 amino acid C-terminal extension [12,21]. It has been shown conceptually that oxyntomodulin reduces bodyweight and food intake [[22], [23], [24]] and increases energy expenditure in humans [25].

At pharmacological doses, glucagon increases energy expenditure, leading to interest in utilizing the actions of glucagon for the treatment of obesity [13,[26], [27], [28], [29], [30], [31]]. However, glucagon is the key counter-regulatory hormone of insulin, and the development of GCGR agonists has been hampered by a glucose-centric perspective on glucagon’s ability to cause hyperglycemia at pharmacological levels. As conceptualized by the pharmacology of oxyntomodulin, the simultaneous activation of the GCGR and GLP-1R shows the therapeutic potential of targeting energy intake and expenditure for weight loss, while maintaining glycemic control [26,32]. The need to appropriately balance the dual mechanism of GCGR/GLP-1R action requires a thorough pharmacological characterization and biomarker strategy for optimal development of unimolecular peptide agonists [[33], [34], [35]].

Here, we describe the discovery and characterization of BI 456906, a once-weekly, injectable GCGR/GLP-1R dual agonist currently in Phase II clinical development for obesity, type 2 diabetes, and NASH. We describe biomarkers that were applied in the discovery of BI 456906 as a balanced dual agonist and demonstrate that engagement of both the GCGR and GLP-1R achieves a bodyweight-lowering efficacy exceeding that of a selective GLP-1R agonist. Applying mRNA sequencing, we provide novel insights into potential mechanisms of action of BI 456906 in hepatocytes, offering a scientific rationale that is supportive of the clinical development for BI 456906 in people with NASH.

## 2 Material and methods

### 2.1 Peptide synthesis

Peptides were synthesized by microwave-assisted solid-phase peptide synthesis (SPPS) using a Fmoc strategy in N-methyl-2-pyrrolidone (NMP) on a polystyrene resin (TentaGel™ S RAM; Rapp Polymere GmbH, Tübingen, Germany). HATU (Hexafluorophosphate Azabenzotriazole Tetramethyl Uronium) was used as coupling reagent together with N,N-diisopropylethylamine as base. Piperidine (20% in NMP) was used for deprotection. The crude peptide was cleaved from resin by treatment with 95/2.5/2.5% (v/v) trifluoroacetic acid (TFA)/triisopropylsilane/water at room temperature for 2 h. After removal of TFA, crude peptides were precipitated and washed with diethyl ether. Peptides were purified by standard high-performance liquid chromatography–mass spectrometry methods and lyophilized.

### 2.2 *In vitro* functional receptor potencies

The functional potencies for of BI 456906 were determined in Chinese hamster ovary (CHO)-K1 cells expressing human GLP-1R and GCGR cDNAs, mouse insulinoma MIN6 cells, and primary human, cynomolgus monkey, mouse, and rat hepatocytes. These assays were applied to support the structure–activity relationship and respective potencies to stimulate cyclic AMP generation, which were determined in the presence of 0.1% BSA. Cells were seeded in 96-well microtiter plates in 100 μL growth medium; growth medium was removed after 24 h (4 h for primary hepatocytes) and the cells were washed with Krebs-Ringer Bicarbonate HEPES buffer (KRBH, 200 μL). The buffer was removed, and the cells were incubated for 15 min at room temperature in 10 μL KRBH (KRBH + 10 mM HEPES, 5 mM NaHCO_3_, 0.1% W/V bovine serum albumin [BSA]) with 0.1 mM 3-isobutyl-1-methylxanthine. The reaction was stopped by the addition of lysis buffer (0.1% W/V BSA, 5 mM HEPES, 0.3% V/V Tween®20) for 10 min at room temperature. Lysates were transferred to 384-well plates and incubated with 10 μL of acceptor/donor bead mixture (AlphaScreen™ cAMP Functional Assay Kit; PerkinElmer, Montreal, Quebec, Canada) for 1 h at room temperature in the dark, then the cAMP content was determined using the AlphaScreen™ cAMP Functional Assay Kit according to the manufacturer’s instructions (PerkinElmer).

To characterize the impact of plasma protein binding for fatty-acid protracted peptides, the cAMP responsive element (CRE)-induced luciferase activity assay was applied, and receptor potencies were determined in the presence of low (0.5%) and high (100%) levels of mouse and human plasma. Human HEK293 CRE-luc2P cells (Promega, Madison, WI, USA) expressing recombinant GLP-1R and GCGR were cultivated in Dulbecco’s Modified Eagle Medium (with high glucose/l-glutamine) supplemented with 10% fetal bovine serum, 50 μg/mL hygromycin, and 400 μg/mL Geneticin™. For the assays, cells were resuspended either in KRBH with 0.5% human or mouse plasma or in 100% human or mouse plasma. Cells were treated for 4 h at 37 °C with the different peptides (all n = 3; all tested peptides were produced in house, handled as 1 mM stock solution in dimethyl sulfoxide, and tested at a final concentration range between 0.2 pM and 1 μM). *In vitro* potency was assessed using the Bright-Glo™ Luciferase Assay System (Promega), measuring the production of cAMP through a CRE-controlled luciferase. The potency (EC_50_), plasma shift, and GLP-1R/GCGR ratio was calculated for each plasma condition.

### 2.3 Inhibition of glycogen synthesis in rat hepatocytes and glucose-stimulated insulin secretion from primary islets *in vitro*

For determining glycogen synthesis inhibition in primary rat hepatocytes, cells were plated in 24-well plates in medium (Williams E Medium [Thermo Fisher Scientific, Waltham, MA, USA] containing 200 mM glutamine, 1 mg/mL gentamicin, dexamethasone 0.1 μM, Insulin-Transferrin-Sodium-Selenite-Supplement [Sigma-Aldrich, St. Louis, MO, USA] 0.017 μM) at 37 °C and 5% CO_2_. After 24 h, the medium was removed, cells were washed once with PBS, and 180 μL KRBH (134 mM NaCl, 3.5 mM KCl, 1.2 mM KH_2_PO_4_, 0.5 mM MgSO_4_, 1.5 mM CaCl_2_, 5 mM NaHCO_3_, 10 mM HEPES, pH 7.4) containing 0.1% BSA, 22.5 mM glucose, the respective peptide concentration, and 40 μCi/mL D-[U-^14^C]-glucose was added. After 180 min, the incubation buffer was aspirated; the cells were washed once with ice-cold PBS and lysed for 30 min at room temperature with 1 mol/l NaOH. Lysates were transferred to 96-well filter plates and glycogen was precipitated by incubation for 120 min at 4 °C followed by washing four times with ice-cold ethanol (70%). The precipitates were filtered to dryness and the synthesized glycogen determined by counting the amount of radioactivity (incorporated ^14^C-glucose) on the filter in a topcount scintillation counter. Each cell plate contained wells with vehicle controls (0.1% DMSO in KRBH) as reference for non-inhibited glycogen synthesis (100% CTL) and wells without D-[U-^14^C]-glucose as control for non-specific background, which was subtracted from all values.

The potencies to increase glucose-stimulated insulin secretion (GSIS) were assessed in mouse and rat islets under static conditions as previously described [36]. Rat and mouse islets were treated with GLP-1, BI 456906, or liraglutide. Untreated cells were exposed to 1.0 mM glucose and compared with those kept under treatment conditions. Glucose was added (8.3 mM for rat islets, 16.7 mM for mouse islets), and insulin secretion was measured (for static GSIS with Insulin High range HTRF kit; Cisbio, Codolet, France; for perifusion experiments with human islets Insulin ELISA kit; ALPCO, Salem, NH, USA) and expressed as a percentage of total insulin.

The Perifusion System (PERI-4.2; Biorep Technologies, Inc., Miami, FL, USA) was used to determine the dynamic insulin secretion of human islets (Prodo Laboratories, Inc., Aliso Viejo, CA, USA) after application of 10 nM BI 456906, 30 nM liraglutide, or buffer (KRBH with 0.1% BSA). Six chambers were used, each containing 50 islet cells. Insulin secretion (ng/mL) was measured over 120 min, with 1 mM glucose applied after 20 min, 8.3 mM glucose after 35 min, 1 mM glucose after 75 min, and 60 mM KCl after 105 min.

### 2.4 Animal studies

Experimental protocols concerning the use of laboratory animals were reviewed by a federal ethics committee and approved by governmental authorities.

Unless otherwise stated, the vehicle used throughout the studies was composed of 50 mM phosphate buffer pH 7.0 and 5% mannitol. All agent dosing was carried out at a volume of 5 mL/kg.

### 2.5 Pharmacokinetic properties of BI 456906

For pharmacokinetic analysis, male C57BL/6NRj mice (Janvier, France) were given a single intravenous (IV) or subcutaneous (SC) BI 456906 dose (20 nmol/kg; N = 3 per route). In addition, male Beagle dogs received an IV or SC dose of 5 nmol/kg or 10 nmol/kg (N = 3) BI 456906, respectively. Plasma samples were generated at different time points post dosing and stored at −20 °C until further analysis. Plasma concentrations of BI 456906 were measured using a liquid chromatography/tandem mass spectrometry (LC/MS/MS) method with a calibration range of 0.5–1000 nM on a QTRAP 6500+ (Sciex, Framingham, MA, USA). Samples were pre-treated with ethanol for protein precipitation before the analysis.

### 2.6 Inhibition of acute food intake in NMRI outbred mice

Three-week-old, male, lean NMRI outbred mice (wild-type [WT] and GLP-1R knockout [KO] [37]) were obtained from Janvier Labs (Le Genest-Saint-Isle, France) and were group-housed four mice per cage on a 12 h/12 h dark/light cycle (lights off at 15:00). The room temperature was controlled at 21 °C ± 1 °C, with 60% ± 20% humidity. Animals had *ad libitum* access to regular rodent chow (KLIBA NAFAG 3430; Granovit AG, Kaiseraugst, Switzerland) and tap water. Six-week-old male mice were randomized into treatment groups based on food intake and bodyweight (n = 8–10 per group). Mice were fasted for 6 h before randomization and SC dosing of BI 456906, semaglutide, or vehicle 1 h before the dark phase. Food intake was measured for 24 h using a fully automated Herdsman-2 food-intake monitoring system (MBRose, Faaborg, Denmark). Blood was drawn at 24 h post dosing, and amino acid levels were measured (see later). ED_50_ values were calculated in GraphPad Prism 9 using the nonlinear fit tool.

### 2.7 Acute glucose tolerance tests in lean mice

The effect of BI 456906 on acute glucose tolerance was assessed by intraperitoneal and oral glucose tolerance tests. Male WT and GLP-1R KO lean C57BL6/J mice (Janvier Labs), 10–12 weeks old, were randomized (n = 7 per group) and fasted for 12 h prior to study initiation. Pre-treatment blood glucose was measured at −5 h. Mice were administered SC BI 456906 or semaglutide at −4 h. Baseline blood glucose was measured at −1 h, and then an intraperitoneal bolus of 2 g/kg glucose was applied at 0 h. Blood glucose was measured at 0, 15, 30, 60, and 120 min, and compound exposure was measured at 140 min.

Male WT and GLP-1R KO lean C57BL6/J mice, 6–7 weeks old (n = 6–7 per group), were administered SC vehicle, BI 456906, or semaglutide at −24 h. Mice were then fasted from −10 h, before bolus ingestion of glucose (2 g/kg) at 0 h. Blood glucose was measured at 0, 15, 30, 60, and 120 min, and compound exposure was measured at 120 min. In both experiments, glucose was measured in whole blood using a commercially available glucometer (GlucoSmart Swing) with test strips (MSP bodmann GmbH, Bobingen, Germany). Data are represented as mean ± standard error of mean (SEM) and were compared using GraphPad Prism 9 using two-way ANOVA, followed by Dunnett’s method for multiple comparisons versus vehicle. Significant differences were identified at p < 0.05.

Area under the curve (AUC) was calculated from glucose concentrations measured between 0 and 120 min. ED_50_ values were calculated in GraphPad Prism 9 using the nonlinear fit tool.

### 2.8 Acute gastric emptying in lean mice

Lean, male, 8–10-week-old B6J mice were fasted for 12 h before study initiation, with SC compound dosing of vehicle, BI 456906, or semaglutide at −4 h. At 0 h, mice had a bolus ingestion of acetaminophen (100 mg/kg)–glucose (2 g/kg), and the acetaminophen concentration was measured by plasma sampling via the vena facialis at 10, 30, and 60 min. After 60 min, *ex vivo* assays assessing acetaminophen, blood glucose, and terminal exposure were carried out. Acetaminophen was measured using an automated analyzer (Cobas Integra 400; Roche, Rotkreuz, Switzerland). AUC was calculated from acetaminophen concentrations measured between 0 and 60 min. ED_50_ values were calculated in GraphPad Prism 9 using the nonlinear fit tool.

### 2.9 Nicotinamide N-methyltransferase gene expression in liver

Male WT C57BL6/J mice, 6–7 weeks old (n = 5 per group), were administered SC vehicle, semaglutide, or BI 456906 at −18 h. Mice were then fasted from −12 h until 0 h, when they were euthanized, a lobe of the liver was preserved in RNA*later*™ Stabilization Solution (Thermo Fisher Scientific) at 4 °C, and nicotinamide N-methyltransferase (NNMT) mRNA expression in the liver was measured by real-time PCR using a TaqMan gene-expression assay (Applied Biosystems, Waltham, MA, USA).

### 2.10 Imaging study in cAMP response element-luciferase transgenic mice

Male cAMP response element (CRE)-luciferase (Luc) transgenic mice (FVB/NTac-Tg [CRE/Tk-luc]187Hgp; Taconic Biosciences, Inc., Rensselaer, NY, USA) were obtained at 12–13 weeks old. Animals were fed irradiated Teklad 2918.15 Rodent Diet (Envigo, IN, USA) and water *ad libitum*. Mice were sorted into study groups (n = 5 per group) based on bodyweight and baseline images obtained on Day −1. All mice were dosed according to individual bodyweight with SC vehicle, semaglutide, BI 456906, or a long-acting glucagon (LA-GCG) analog (each 100 nmol/kg) or dosed with isoproterenol by intraperitoneal injection (10 mg/kg). *In vivo* bioluminescence imaging was carried out at Day −1 (pre-dose) and Day 0 (4, 8, and 12 h post dose). Bioluminescence imaging was performed using an IVIS Spectrum (PerkinElmer, Inc., Waltham, MA, USA) under 1–2% isoflurane gas anesthesia. Mice were injected subcutaneously at the base of the neck with 250 mg/kg (25 mg/mL) D-luciferin and imaged in the prone, supine, and left lateral positions 10 min after injection. Images were analyzed using Living Image software (version 4.7.1; PerkinElmer). At 12 h post dose following the last imaging time point, all mice were euthanized via overexposure to CO_2_ for blood and tissue collection. Whole blood was collected via cardiac puncture and used to generate plasma. For dose–response effects of respective compounds, luciferase activity was measured *ex vivo*. Here, mice were dosed according to individual bodyweight with SC vehicle, semaglutide, BI 456906, or LA-GCG. After 4 h, liver and pancreas were harvested and homogenized. Luciferase activity was measured using the luciferase assay system (Promega) [38].

### 2.11 Biomarker study in mice with diet-induced obesity

Male C57BL6/J mice pre-fed with a 60% high-fat diet (HFD) were obtained from The Jackson Laboratories (Bar Harbor, ME, USA) at >16 weeks of age (n = 11 per group). Animals were housed with a 10:00–22:00 dark/light cycle throughout. For 10 days, the mice were given chronic repeated SC compound dosing at 08:00 with vehicle (twice daily [BID]), BI 456906 (7.5 nmol/kg once daily [QD]), semaglutide (10 nmol/kg QD), or LA-GCG (30 nmol/kg BID), with bodyweight and food intake measured every day. On treatment Days 1, 3, 5, 8, and 10, blood was drawn at the same time each day, and blood glucose (n = 11) and plasma active ghrelin and glucagon (n = 6) were measured. Data are represented as mean ± SEM and were compared using GraphPad Prism 9 using two-way ANOVA followed by Dunnett’s method for multiple comparisons versus vehicle. Significant differences were identified at p < 0.05.

### 2.12 Energy expenditure study in mice with diet-induced obesity

Male C57BL/6J mice were obtained from Charles River Laboratories (Sulzfeld, Germany) at age 6–7 weeks. Animals were housed in groups of five in individually ventilated cages with a 12 h light/dark cycle (06:00–18:00) and were acclimatized for 1 week with *ad libitum* feeding. Animals were then fed a 45% HFD for 18 or 30 weeks, before randomization to groups with similar average bodyweight at Day −1. Animals were dosed QD with SC vehicle (25 mM phosphate-buffered saline) or BI 456906 (5, 10, or 20 nmol/kg) for 9 days, with energy metabolism, activity, and core body temperature measured each day. On Day 10, mice were sacrificed by cervical dislocation after a final bleeding by puncture of the retrobulbar venous plexus.

Energy metabolism was measured by indirect calorimetry. Mice were acclimatized to housing in respiratory cages inside a climate chamber for 2 days prior to compound dosing. The mass flow of air through the respiratory cages and the concentrations of O_2_ and CO_2_ in inspired and expired air were measured every 10 min. These were used to calculate the respiratory quotient and energy expenditure (according to Heldmaier [39]). Energy expenditure was analyzed by ANCOVA (see 2.16) for animals kept in single housing (dose groups 5 and 10 nmol/kg), applying statistical analysis using a general linear model framework including bodyweight as a covariate [40]. Because animals in the 20 nmol/kg dose group were kept in group housing only, mean values of energy expenditure without animal-to-animal variability were not available. Therefore, the regression-based ANCOVA was not calculated. Core body temperature and activity were measured by sensors (model 10TA10TA-F10; Data Sciences International, New Brighton, MA, USA) implanted in the peritoneal cavities. Transmitters were implanted 1 week before acclimatization to the respiratory cages. Data acquisition and analysis were carried out by DQUART software (Data Sciences International).

### 2.13 Subchronic, repeated dosing study in diet-induced obese mice

Male C57BL6/J mice pre-fed with a 60% HFD were obtained from The Jackson Laboratories at >16 weeks of age. Upon arrival, mice were single-housed to obtain accurate and individual food intake measurements for each animal. During the entire study, animals had *ad libitum* access to food (HFD D12492; Research Diets, Inc., New Brunswick, NJ, USA) and tap water. Before the start of the study, animals were randomized based on their bodyweight measured 1 week prior to the start of treatment (n = 11 per group). At study start, the age of the mice was 22 weeks. Mice were administered chronic repeated SC dosing of vehicle, BI 456906 (3, 10, 20, or 30 nmol/kg), or semaglutide (20 or 100 nmol/kg) daily for 30 days, with bodyweight and food intake measured every day. At Day 29, EchoMRI™ 4in1-900 (EchoMRI, Houston, TX, USA) was carried out. At study termination (Day 30), exposure was measured at 7 h (C_max_) and 24 h (C_min_) post dose, along with multiple biochemistry markers. Blood was drawn, and plasma triglycerides, cholesterol, free fatty acids, aspartate aminotransferase (AST), alanine aminotransferase (ALT), glucagon, fibroblast growth factor-21 (FGF-21), amino acids, and *ex vivo* bioactivity were measured. The liver was homogenized, and concentrations of triglycerides and cholesterol were measured. Plasma and liver triglycerides, cholesterol, plasma amylase, and lipase were measured using an automated analyzer (Cobas Integra 400; Roche). Plasma glucagon and FGF-21 were measured using an enzyme-linked immunosorbent assay and colorimetric kits (MSD® Metabolic Panel; Meso Scale Diagnostics, Rockville, MD, USA). Insulin, leptin, and active ghrelin were measured using a MILLIPLEX® Mouse Metabolic Hormone Multiplex Assay (Merck KGaA, Darmstadt, Germany). An enzyme-linked immunosorbent and colorimetric assay kit was used to measure free fatty acids (Wako Pure Chemicals, Osaka, Japan) in EDTA plasma.

To determine the activity of semaglutide and BI 456906, heparin plasma was derived from euthanized animals. The plasma was diluted in 100% mouse plasma, and the respective concentrations to induce luciferase activities were determined *in vitro* using human embryonic kidney cells expressing recombinant human GLP-1R and human GCGR and the CRE-inducible luciferase. A standard curve in 100% mouse plasma was generated with human GLP-1-(7–36) amide (containing 1 μM linagliptin) and human glucagon. The bioactivity of semaglutide and BI 456906 *ex vivo* was calculated for the GLP-1R and GCGR for each individual sample and animal based on the standard curves for GLP-1 and glucagon, calculated as relative activity over the actual potency (EC_50_) for each molecule and dose and displayed in relation to the exposure of the molecule. For BI 456906, a relative potency ratio *ex vivo* for the GLP-1R and GCGR was derived from the activity of endogenous GLP-1 and glucagon observed *in vitro*.

### 2.14 Liver RNA sequencing

Total RNAs extraction was performed as previously described [41]; 5 mg of tissue was placed in the lysis solution and homogenized in Tissuelyser™ (Qiagen, Hilden, Germany) for 30 s. Total RNA samples were quantitatively and qualitatively assessed using the fluorescence Broad Range Quant-iT RNA Assay Kit (Thermo Fisher Scientific) and the Standard Sensitivity RNA Analysis DNF-471 Kit on a 96-channel Fragment Analyzer (Agilent Technologies, Santa Clara, CA, USA), respectively. All total RNA samples had an RNA integrity number >8. Total RNA input of 250 ng was employed for library preparation with the NEBNext Ultra II Directional RNA Library Prep Kit for Illumina #E7760, together with the NEBNext Poly(A) mRNA Magnetic Isolation Module #E7490 and NEBNext Multiplex Oligos for Illumina #E7600 (New England Biolabs, Ipswich, MA, USA) as per manufacturer’s instructions. AMPure XP beads (Beckman Coulter) for double-stranded cDNA purification were used. Libraries were amplified with 12 PCR cycles. The final mRNA libraries were eluted in 30 μL EB Buffer and were quantified by the High Sensitivity dsDNA Quant-iT Assay Kit (Thermo Fisher Scientific) on a Synergy HTX (BioTek). Libraries were assessed for size distribution and adapter dimer presence (<0.5%) by the High Sensitivity Small Fragment DNF-477 Kit on a 96-channel Fragment Analyzer (Agilent). Sequencing libraries were normalized on the Microlab STAR (Hamilton), pooled and subsequently clustered on an S2 Flow Cell and sequenced on a NovaSeq 6000 Sequencing System (Illumina) with dual index, paired-end reads at 2 × 100 bp length with an average sequencing depth of 55 million Pass-Filter reads per sample.

Sequencing reads from the RNA-Seq experiment were processed as previously described [42]. Differential expression analysis was performed on the mapped counts derived from featureCount using limma/voom [43,44]. If not otherwise stated, an absolute log2 fold change cut-off of 1 and a false discovery rate (FDR) of ≤0.05 was used for further analysis. BI 456906-specific dose pattern differentially expressed genes were selected based on (i) log2 fold change 1, FDR<0.05, between control–vehicle versus 30 nmol/kg BI 456906 and semaglutide versus 30 nmol/kg BI 456906; and (ii) no significant difference between control–vehicle versus semaglutide. Gene expression patterns for differentially expressed genes were computed and visualized using the DEG report R package (version 1.28.0) [45]. Clusters of genes from a previous study by Pantano et al. on human non-alcoholic fatty liver disease samples [46] from subjects at different fibrosis stages were used to compare gene expression patterns against the current study.

### 2.15 Plasma amino acid measurements with liquid chromatography/tandem mass spectrometry

Plasma samples were thawed and diluted 20 × with water (5 μL plasma +95 μL H_2_O), mixed, and centrifuged for 1 min at 4000 rpm. A total of 20 μL of the supernatant was transferred to a new well containing 5 μL of amino acid internal standard (IS). Finally, a volume of 225 μL of acetonitrile/water (90/10 v:v) was added to each sample. The LC-MS/MS analysis was carried out using the following conditions: for liquid chromatography, mobile phase A comprised 100 mM ammonium formate and 0.2% formic acid, and mobile phase B comprised acetonitrile and 0.2% formic acid. Analytes were separated with a Poroshell 120 HILIC-Z, 2.1 × 100 mm, 2.7 μm (Agilent) using Agilent 1290 Infinity II LC with a gradient mode. Following the separation, amino acid analyses were conducted with a QTRAP 6500+ triple-quadrupole mass spectrometer (Sciex, Darmstadt, Germany), operated in positive electrospray ionization mode, using specific multiple reaction monitoring methods for each of the amino acids and their ^13^C-labeled IS (alanine, arginine, citrulline, glutamine, glycine, isoleucine, leucine, lysine, ornithine, proline, serine, threonine, tyrosine, valine). Stock solutions of the ISs were prepared in 0.1 M HCl solution. Quality control samples were prepared by spiking individual working solutions into blank matrix or surrogate matrix. The Analyst software (Sciex, Germany) was used for results analysis.

### 2.16 Statistical analysis

Unless stated earlier, data are presented as mean ± SEM and were compared using one-way ANOVA followed by Dunnett’s method for multiple comparisons versus vehicle. Comparisons were considered significant at p < 0.05. Analyses were performed using GraphPad 9 statistical software (GraphPad Software). Energy expenditure measurements were analyzed by ANCOVA using SAS (Statistical Analysis Software) version 9.4. and performed separately per day using a generalized linear model. This involves the “baseline observation” at Day 1 prior to dosing and the covariate “bodyweight” to compare different dose levels of the test item to negative control. Degrees of freedom were calculated according to Kenward and Roger and one-sided pairwise comparisons with negative control were performed using a 5% level of significance.

## 3 Results

### 3.1 Chemical structure of BI 456906

**Figure 1:**
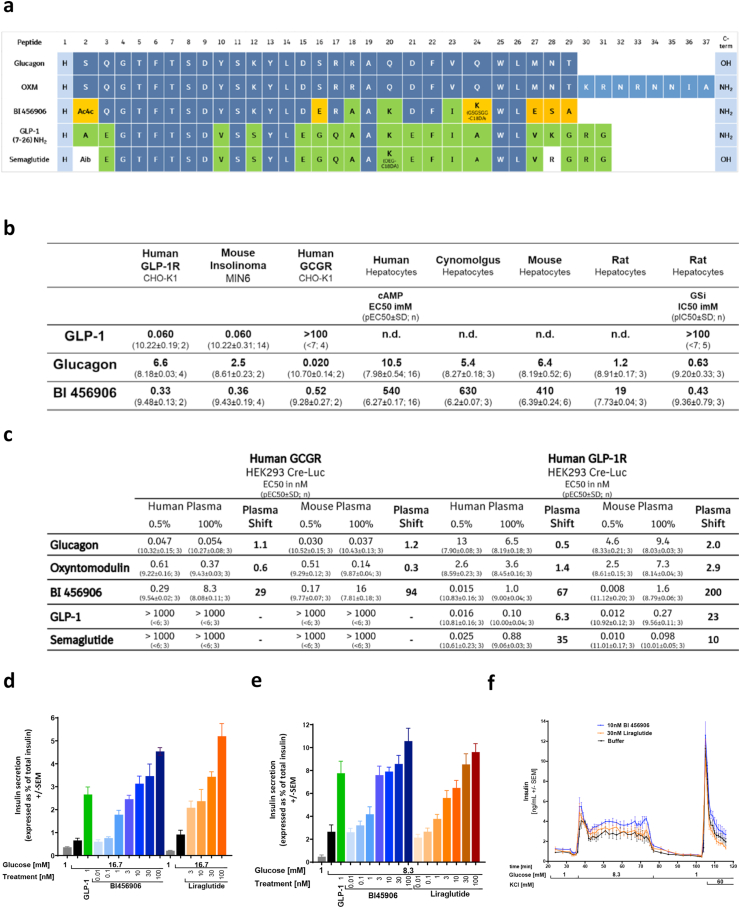
**Structural properties and *in vitro* profile of BI 456906.** (a) BI 456906 was designed based on a modified glucagon sequence, incorporating an unnatural amino acid and a glycine–serine linker in position 24, containing a C18 di-acid. (b) BI 456906 potency at the human GCGR and GLP-1R to stimulate cAMP in cells with recombinant (CHO-K1) and endogenous (MIN6, hepatocytes) receptor expression. The functional potency at the GCGR was confirmed by inhibition of glycogen synthesis in rat hepatocytes. (c) Potency of BI 456906, glucagon, GLP-1, oxyntomodulin and semaglutide at the human GCGR and GLP-1R based on the luciferase induction in CRE-Luc cells in the presence of 0.5% and 100% mouse or human plasma. (d–f) Glucose-stimulated insulin secretion after treatment with GLP-1 (1 nM), BI 456906, or liraglutide (0.01, 0.1, 1, 3, 10, 30, or 100 nM) in (d) mouse, (e) rat, and (f) perifused human pancreatic islets. Results are shown as insulin secretion expressed as a percentage of total insulin ±SEM. CRE-Luc, cAMP response element-luciferase; EC50, half maximal effective concentration; GCGR, glucagon receptor; GLP-1, glucagon-like peptide-1; GLP-1R, glucagon-like peptide-1 receptor; IC50, half maximal inhibitory concentration; OXM, oxyntomodulin; pEC50, negative log EC50; pIC50, negative log IC50.

BI 456906 is a 29-amino-acid peptide optimized to balance the degree of agonism for the human GCGR and GLP-1R by introducing various modifications to the human glucagon sequence (Figure 1A). Proteolytic stability of BI 456906 is helped by C-terminal amidation and the introduction of a non-coded amino acid 1-aminocyclobutane-1-carboxylic acid (Ac4c) in position 2, well established as the site of proteolytic activity for dipeptidyl peptidase-4. The desired extended terminal half-life of BI 456906 was achieved by the introduction of a glycine–serine linker in position 24, carrying a C18 di-acid. This acylation of BI 456906 mediates albumin binding to increase the terminal half-life. Upon SC injection of BI 456906 in preclinical species, mean residence times of 44 and 140 h and T_max_ values of 7 and 51 h were obtained in mice and dogs, respectively (Table S1), enabling a once-weekly dosing frequency in humans [47]. All relevant structural features of the molecule were subject to optimization to derive BI 456906 as a preferred GCGR/GLP-1R dual agonist with the desired profile: the amino acid sequence (including the structure of the amino acid in position 2), the position and type of linker, and the fatty acid as the half-life extending principle (Figure 1A).

### 3.2 BI 456906 is a potent, selective, GCGR/GLP-1R dual agonist *in vitro*

To establish the effect of peptide modifications on the binding of BI 456906 to GCGRs and GLP-1Rs, we applied a series of functional assays to characterize BI 456906 *in vitro*. The functional potency (EC_50_) of BI 456906 in CHO-K1 cells was 0.33 and 0.52 nM for GLP-1R and GCGR, respectively, showing an approximately 10-fold lower potency compared with the native ligands GLP-1 (60 pM) and glucagon (20 pM) (Figure 1B). The EC_50_ for the endogenous mouse GLP-1R was assessed in the insulinoma cell line MIN6 (Figure 1B); the EC_50_ was 0.36 nM for BI 456906 and 60 pM for GLP-1. The ability of BI 456906 to activate the endogenous GCGR was determined in primary mouse, rat, cynomolgus monkey, and human hepatocytes, based on cumulative measurements of cAMP and functional inhibition of glycogen synthesis in primary rat hepatocytes (Figure 1B).

The impact of the albumin binding half-life extension on the receptor potencies for BI 456906 was characterized in the presence of 0.5% or 100% human or mouse plasma and compared with endogenous receptor ligands and other incretin agonists (Figure 1C). In 0.5% human and mouse plasma, BI 456906 showed a similar potency to that of endogenous GLP-1. For the GCGR, in 0.5% human and mouse plasma, BI 456906 was ∼6-fold less potent (0.29 and 0.17 nM, respectively) in relation to endogenous glucagon (47 and 30 pM, respectively) (Figure 1C). When the potency was assessed in the presence of 100% human and mouse plasma, BI 456906 showed a potency of 1.0 and 1.6 nM for the human GLP-1R and 8.3 and 16 nM for the human GCGR, respectively. The shift in potency was higher in 100% mouse plasma compared with 100% human plasma, suggesting a stronger affinity of BI 456906 to mouse plasma proteins compared with human. The increased affinity to mouse plasma proteins is supported by the pharmacokinetic profiles of BI 456906 and the long-acting GLP-1R agonist semaglutide in mouse plasma, with a longer terminal half-life seen for BI 456906 compared with semaglutide (Table S1).

The functional relevance of the effects of BI 456906 was confirmed for potentiating GSIS in mouse and rat pancreatic islets, which was similar to native GLP-1 and the long-acting GLP-1R agonist liraglutide (Figures 1D, E, S1a and b). In perifused human islets, BI 456906 showed a first- and second-phase insulin-secretory response that was similar to that of liraglutide (Figure 1F).

### 3.3 BI 456906 engages the glucagon-like peptide-1 receptor *in vivo* upon single dosing to reduce food intake, improve glucose tolerance, and inhibit gastric emptying

**Figure 2:**
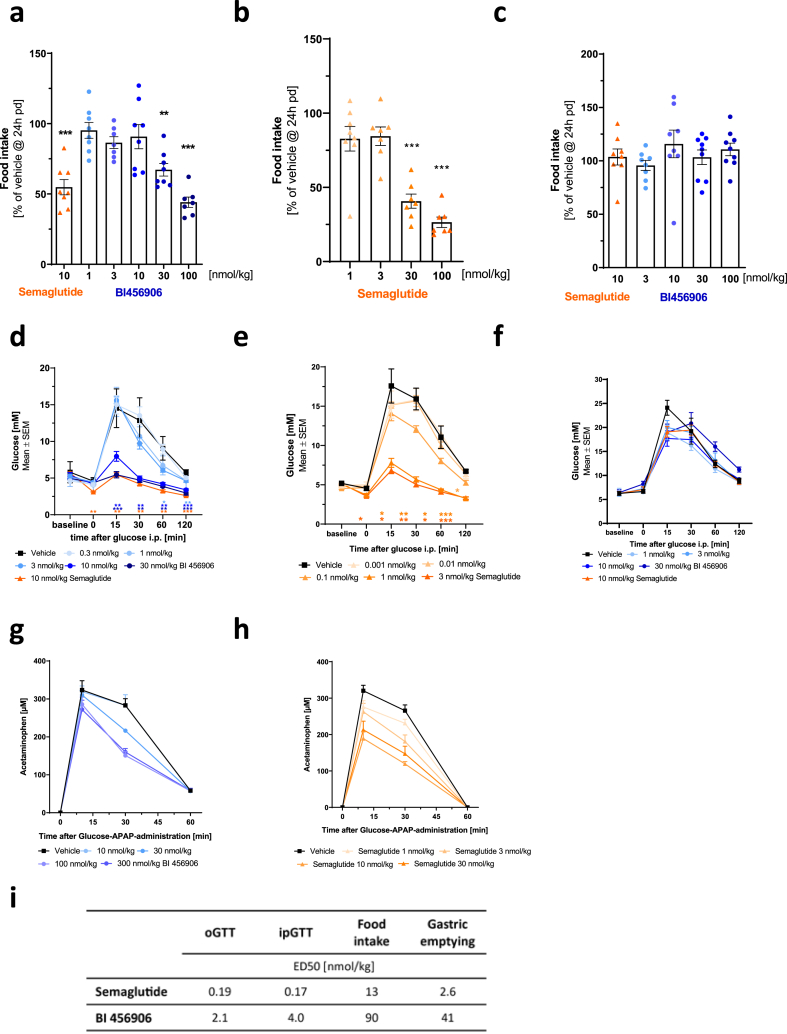
**Glucagon-like peptide-1 receptor engagement of BI 456906 to reduce food intake and gastric emptying and to improve glucose tolerance in lean animals after single dosing.** The effects of BI 456906 and semaglutide are shown on food intake (a–c), glucose tolerance (d–f), and gastric emptying (g and h). Food intake is shown as percentage of vehicle food intake 24 h after dosing with (a) BI 456906 or (b) semaglutide in wild-type mice, and (c) in GLP-1R knockout mice. Glucose tolerance tested by intraperitoneal glucose administration after dosing with (d) BI 456906 or (e) semaglutide in wild-type mice and (f) in GLP-1R knockout mice. Gastric emptying was tested by acetaminophen concentration after ingestion of an acetaminophen-glucose bolus in wild-type mice after dosing with (g) semaglutide and h) BI 456906. (i) Summary of acute potencies (ED_50_) of BI 456906 compared with semaglutide. Data are shown as mean ± SEM. Statistical analysis was done using one-way ANOVA followed by Dunnett’s method for multiple comparisons versus vehicle with significance defined at ∗p < 0.05, ∗∗p < 0.01, ∗∗∗p < 0.001. APAP, acetaminophen; GLP-1R, glucagon-like peptide-1 receptor; iGTT, intraperitoneal glucose tolerance test; oGTT, oral glucose tolerance test.

The acute potency of BI 456906 in engaging the GLP-1R was tested in lean mice after single dosing; the ability of BI 456906 to reduce food intake, improve glucose tolerance, and inhibit gastric emptying was compared with semaglutide. Acute food intake was dose-dependently reduced by BI 456906 (Figure 2A) and semaglutide (Figure 2B) in WT but not in GLP-1R KO mice (Figure 2C), showing a lower potency for BI 456906 (Figure 2I). Similarly, both BI 456906 and semaglutide improved intraperitoneal and oral glucose tolerance in WT (Figures 2D, E, and S2) but not in GLP-1R KO mice (intraperitoneal, Figures 2F, S2c; oral, Figure S2h). Reduced acetaminophen excursion, a measure for inhibition of gastric emptying, was observed for both BI456906 (Figure 2G) and semaglutide (Figure 2H), with a higher potency for semaglutide compared to BI 456906, which was effective only at higher doses of 100 and 300 nmol/kg.

Overall, the efficacy of BI 456906 to reduce food intake, improve glucose tolerance, and delay gastric emptying was similar to that of semaglutide, but with an approximately 10- to 20-fold lower potency at the GLP-1R (Figure 2I). This is in concordance with the lower *in vitro* potency of BI 456906 compared with semaglutide determined for the human GLP-1R and GCGR in CRE-Luc cells in 100% mouse plasma (1.6 nM compared with 0.098 nM; Figure 1C), suggesting that plasma protein binding is notably higher for BI 456906 in mouse plasma.

### 3.4 BI 456906 achieves a greater bodyweight-lowering efficacy in diet-induced obese mice compared with maximally effective doses of semaglutide

**Figure 3:**
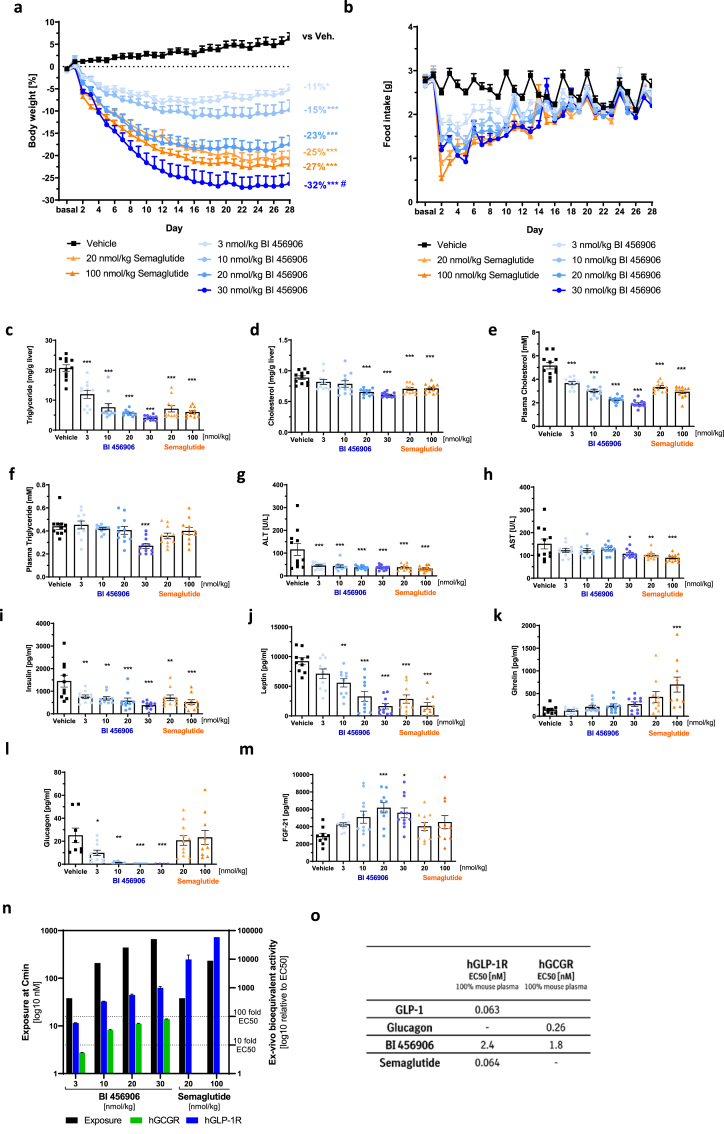
**BI 456906 demonstrates significantly greater bodyweight-lowering efficacy compared with maximally effective doses of semaglutide in diet-induced obese mice by engaging the glucagon receptor.** Mice received repeated SC dosing of vehicle, semaglutide, or BI 456906 for 30 days. (a) Percentage change in bodyweight from baseline to Day 28. (b) Effects of BI 456906 and semaglutide vs vehicle on food intake to Day 28. Concentrations of (c) liver triglycerides, d) liver cholesterol, (e) plasma cholesterol, (f) plasma triglycerides, (g) plasma ALT, and (h) plasma AST at Day 28. Plasma levels of (i) ghrelin, (j) insulin, (k) leptin, (l) glucagon, and m) FGF-21 at Day 28. (n) The nM exposures and *ex vivo* bioactivity of BI 456906 and semaglutide displayed as relative nM activity based on (o) the EC_50_ for the human GLP-1R and GCGR determined in 100% mouse plasma. Data are shown as mean ± SEM, with individual data shown. Statistical analysis was done using one-way ANOVA followed by Dunnett’s method for multiple comparisons versus vehicle with significance defined at ∗p < 0.05, ∗∗p < 0.01, ∗∗∗p < 0.001 vs vehicle; ^#^p < 0.05 vs 20 nmol/kg semaglutide. ALT, alanine aminotransferase; AST, aspartate aminotransferase; FGF-21, fibroblast growth factor-21; GCGR, glucagon receptor; GLP-1, glucagon-like peptide-1; GLP-1R, glucagon-like peptide-1 receptor; hGCGR, human glucagon receptor; hGLP-1R, human glucagon-like peptide-1 receptor; SC, subcutaneous.

Upon repeated QD SC administration of BI 456906 or semaglutide to DIO mice, BI 456906 dose-dependently reduced bodyweight from baseline by up to 32% at Day 28 at a dose of 30 nmol/kg (Figure 3A). The efficacy of 30 nmol/kg BI 456906 to reduce bodyweight was greater than semaglutide at maximally effective doses of 20 nmol/kg (25%) and 100 nmol/kg (27%), the difference reaching significance compared with the 20 nmol/kg dose but not 100 nmol/kg. The marked reductions in bodyweight upon treatment with BI 456906 and semaglutide were reflected by an immediate suppression of food intake compared with vehicle-treated mice, with a maximal anorectic effect seen between Days 1 and 2 (Figure 3B). However, the acute food intake reduction of BI 456906 was less pronounced compared with semaglutide, despite equimolar doses, suggesting an additional mechanism driving the superior weight loss efficacy after 28 days of treatment. Food intake gradually returned to baseline levels and began to normalize around Day 15 for both BI 456906 and semaglutide.

BI 456906 and semaglutide dose-dependently decreased fat mass, with significant reductions seen with doses of 10, 20, and 30 nmol/kg and 20 and 100 nmol/kg for the two compounds, respectively (Figure S3a). Lean mass was significantly reduced by BI 456906 at doses of 20 and 30 nmol/kg and semaglutide at doses of 20 nmol/kg, respectively (Figure S3b). The dose-dependent lowering of liver triglycerides and both liver and plasma cholesterol by BI 456906 (versus vehicle) was similar to the effects seen with semaglutide (Figure 3C–E). Plasma triglycerides were significantly reduced by 30 nmol/kg BI 456906 versus vehicle (Figure 3F). Relative to vehicle, concentrations of ALT were significantly reduced by BI 456906 and semaglutide at all doses investigated, and AST concentrations were significantly reduced by 30 nmol/kg BI 456906 and 20 nmol/kg and 100 nmol/kg semaglutide (Figure 3G and H). Despite lesser reductions in bodyweight compared with 20 nmol/kg semaglutide, doses of 3 and 10 nmol/kg BI 456906 significantly reduced liver triglycerides, plasma cholesterol, and ALT levels (Figure 3C, E, and g). Furthermore, BI 456906 dose-dependently and significantly decreased plasma insulin and leptin versus vehicle (Figure 3I and J), similar to semaglutide. At the end of the study, when food intake had returned to levels in vehicle-treated animals, 100 nmol/kg semaglutide-treated mice showed significantly increased plasma levels of the orexigenic hormone ghrelin versus vehicle, a non-significant trend that was also observed with increasing BI 456906 doses (Figure 3K).

BI 456906 dose-dependently reduced plasma glucagon, an effect that was not observed with semaglutide (Figure 3L). Engagement of the GCGR by BI 456906 was supported by dose-dependent increases in plasma FGF-21 (Figure 3M).

Upon termination of the study, an *ex vivo* bioactivity assay [[48], [49], [50]] was carried out on plasma samples to determine drug exposures and the relative bioactivity for the human GLP-1R and GCGR (Figure 3N; Table S2) to support the interpretation of the pharmacokinetic and pharmacodynamic profiles of the two peptides (Table S1). Diluted plasma samples were used to determine the nM activity and relative bioactive fraction (EC_50_) for BI 456906 and semaglutide at the GLP-1R (Figure S4) and the GCGR (Figure S5). As illustrated in Figure 3O, the *ex vivo* bioactivity allowed the determination of the bioactivities for the respective receptors in relation to the treatment exposure (Table S2); the relative bioactivity of BI 456906 at the GLP-1R was lower compared with semaglutide (Figure 3O). The *ex vivo* activity of semaglutide in plasma exceeded its EC_50_ in 100% mouse plasma more than 1000-fold, suggesting full engagement of the GLP-1R at both doses investigated (Figure 3N and O). BI 456906, at its maximal dose of 30 nmol/kg, showed a bioequivalence of less than 1000-fold and 100-fold for the GLP-1R and GCGR, respectively (Figure 3N). Taken together, these analyses support the conclusion that, unlike semaglutide, the bodyweight-lowering efficacy of BI 456906 is related to engagement of both receptors.

### 3.5 BI 456906 lowers bodyweight while maintaining glycemic control and increasing energy expenditure in mice with diet-induced obesity

**Figure 4:**
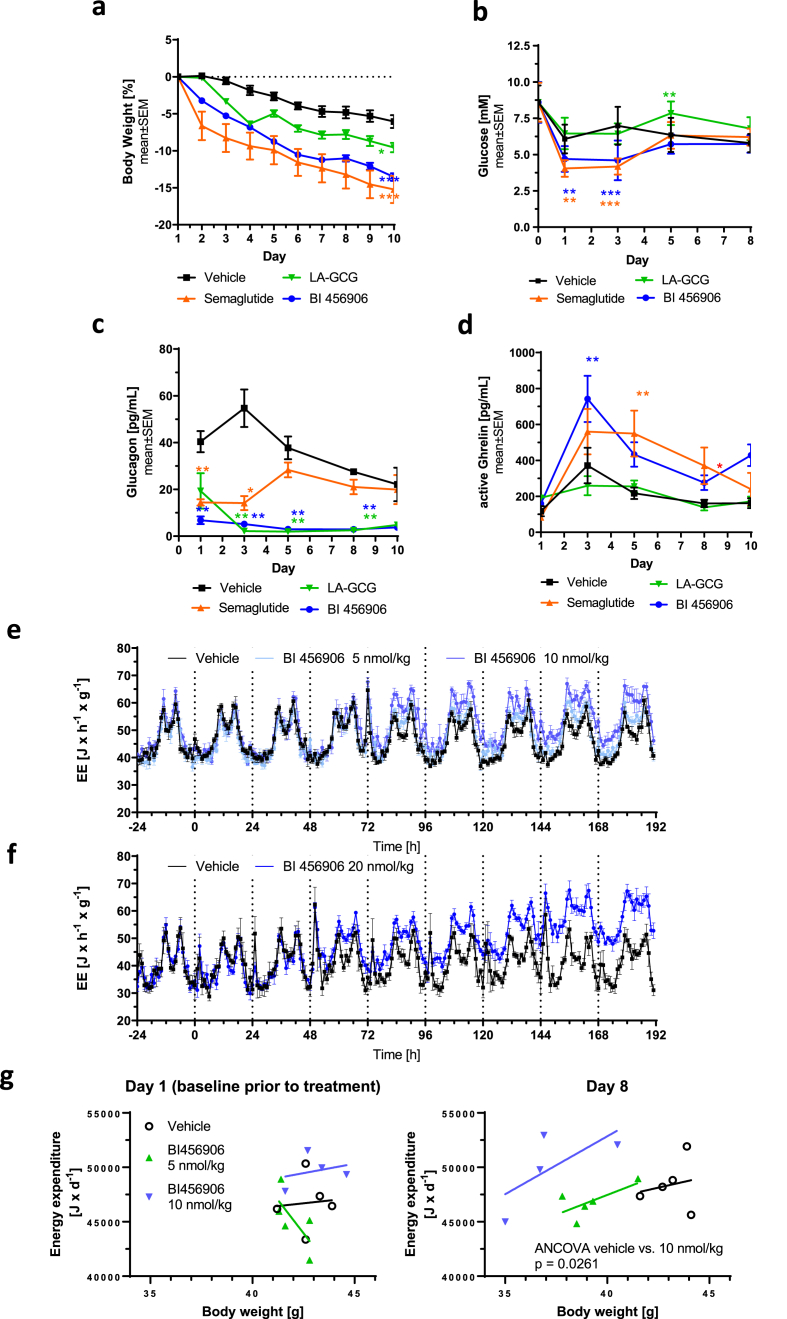
**Effects of BI 456906 on glycemia and energy expenditure in diet-induced obese mice.** DIO mice received chronic repeated dosing of vehicle (BID), BI 456906 (QD), semaglutide (QD), or LA-GCG (BID) for 10 days. (a) Change in bodyweight from baseline. Plasma levels of (b) glucose, (c) glucagon, and (d) active ghrelin over 10 days of treatment. Energy expenditure over 10 days in DIO mice treated with daily dosing of (e) 5 or 10 nmol/kg BI 456906 or (f) 20 nmol/kg BI 456906. (g) Data in panel e have been analyzed separately per day using a general linear model with energy expenditure as a dependent variable, a baseline observation (Day 1 measurement prior to dosing), treatment as a fixed factor, and the covariate bodyweight to compare different dose levels of the test item with negative control (vehicle). Degrees of freedom were calculated according to Kenward and Roger, and one-sided pairwise comparisons with vehicle were performed using a 5% level of significance. Significance level at Day 8 represents the treatment effect of the BI 456906 10 nmol/kg group from the ANCOVA analysis. BID, twice daily; DIO, diet-induced obese; EE, energy expenditure; LA-GCG, long-acting glucagon; QD, once daily.

To demonstrate that weight-lowering effects of BI 456906 in DIO mice can be achieved without compromising glycemic control, a subchronic dosing study was performed comparing BI 456906, semaglutide, and LA-GCG at doses expected to yield similar weight reductions after 10 days of treatment. BI 456906 decreased bodyweight from baseline versus vehicle (Figure 4A) to a similar level as semaglutide (Figure 4A), while LA-GCG achieved significant (Figure 4A), yet less extensive, bodyweight reductions versus vehicle. Both BI 456906 and semaglutide significantly decreased blood glucose at Days 1 and 3 versus vehicle, whereas LA-GCG significantly increased blood glucose at Day 5 (Figure 4B). Lowering of plasma glucagon was observed with BI 456906 and LA-GCG up to Day 8 compared with vehicle while semaglutide led to an initial drop in glucagon, returning to vehicle levels after subchronic dosing (Figure 4C). Plasma acyl ghrelin levels were unchanged by treatment with LA-GCG but were significantly increased versus vehicle by BI 456906 and semaglutide at Days 3 and 5, respectively (Figure 4D).

The hypothesis that GCGR agonism drives energy expenditure was investigated in DIO mice. Daily dosing of BI 456906 induced a dose-dependent increase in energy expenditure (Figure 4E and F). The ANCOVA based statistical analysis [98] performed for single-housed animals (Figure 4E and section 2.12) showed a body weight-independent increase of absolute energy expenditure per animal which reached statistical significance for the 10 nmol/kg dose group at day 7 (p = 0.0312) and 8 (p = 0.0261). Treatment with therapeutic doses of BI 456906 did not increase core body temperature or activity of the animals (Figure S6a and b).

### 3.6 BI 456906 drives transcriptional changes in hepatocytes, providing novel mechanistic insights into glucagon receptor agonism

**Figure 5:**
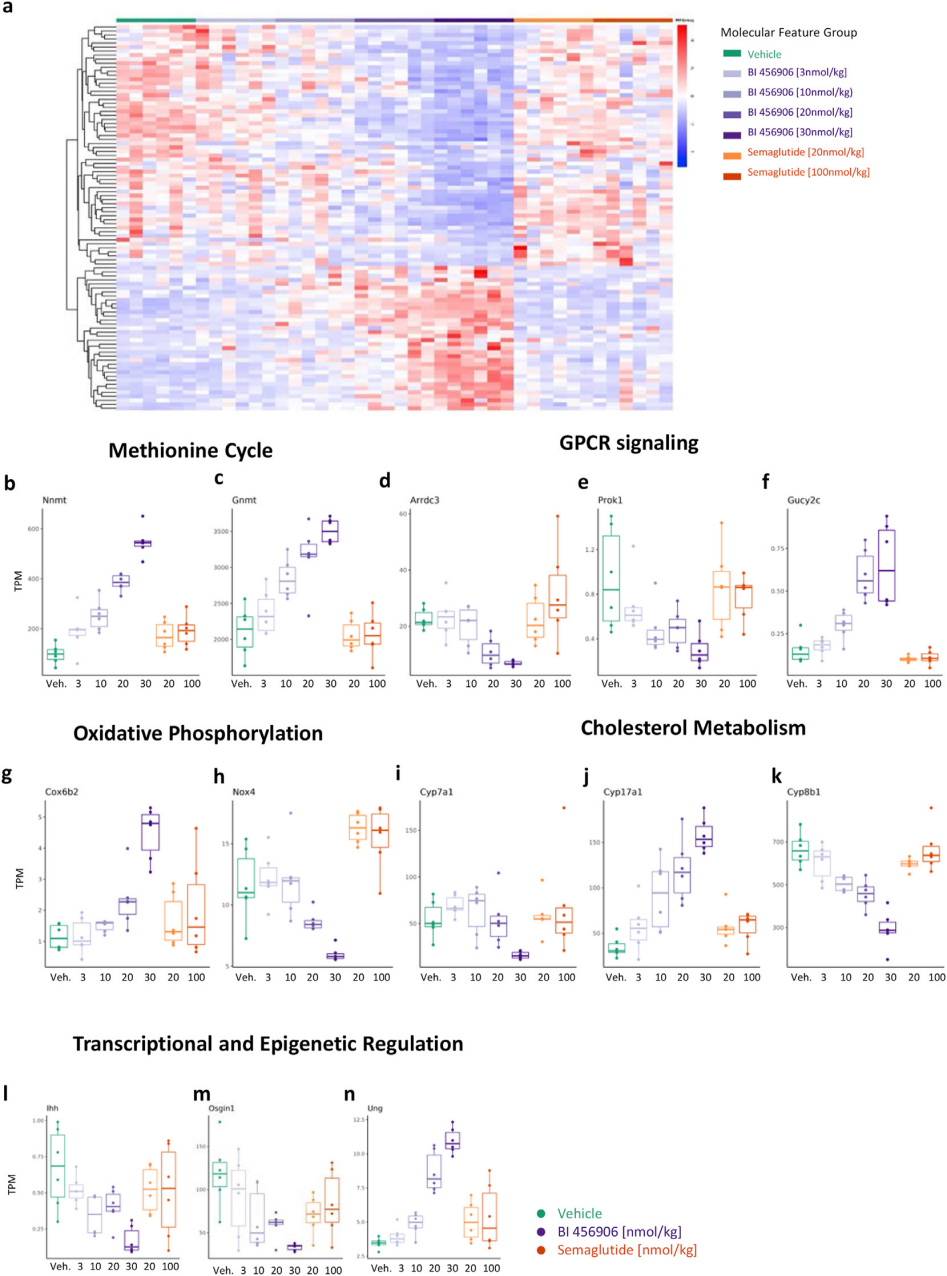
**Modulation of gene expression after dosing with BI 456906 and semaglutide.** Dose-dependent gene expression modulation by BI 456906. (a) Heat map of differential gene expression (93 gene set; log2 fold change >1 or <−1, and FDR <0.05) based on different levels of BI 456906 (3, 10, 20, and 30 nmol/kg) vs the control vehicle, semaglutide (20 and 100 nmol/kg) vs BI 456906 30 nmol/kg, and the control vehicle alone. Each row represents a gene, each column represents a sample, and the color code shows the dose-dependent Z-score of TPM (deviation from a gene’s mean expression in standard deviation units). (b–n) BI 456906 compound-specific dose-dependent effect on genes involved in (b and c) the methionine (GCGR biomarkers), (c–f) GPCR signaling, (g and h) oxidative phosphorylation, (i–k) cholesterol metabolism, and (l–n) cell cycle and differentiation. The middle line denotes median TPM. FDR, false discovery rate; GCGR, glucagon receptor; GPCR, G protein-coupled receptor; TPM, transcripts per million.

The livers from the study represented in Figure 3 were subjected to bulk mRNA sequencing to identify genes that were dose-dependently regulated by BI 456906, but not semaglutide, and therefore not considered secondary to the degree of weight loss. In total, 93 mRNAs fulfilled these criteria (Figures 5A and S7). When grouped by their functional and biochemical relevance, transcriptional changes were seen for mRNAs associated with the methionine cycle (Figure 5B and C), G protein-coupled receptor (GPCR) signaling (Figure 5D–F), oxidative phosphorylation (Figure 5G and H), cholesterol metabolism (Figure 5I–K), and cell cycle and differentiation (Figure 5L–N).

**Figure 6:**
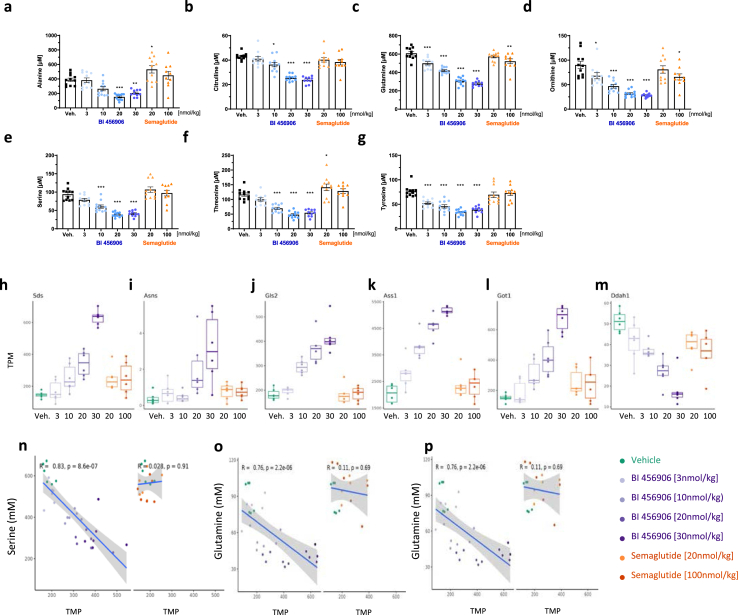
**Plasma and transcriptional markers of glucagon receptor activation by BI 456906.** Amino acid regulation by BI 456906. (a–g) Plasma amino acid levels at Day 28 after subchronic dosing with vehicle, BI 456906, or semaglutide in DIO mice. Data are shown as mean ± SEM, with individual data shown. (h–m) The dose-dependent regulation of genes encoding amino acid-metabolizing enzymes by BI 456906 versus semaglutide. The middle line denotes median transcripts per million (TPM) (n–p) Correlation of transcriptional changes in *Sds* (n), *Gls2* (o), and *Got1* (p) mRNA with plasma levels of serine (*Sds*) and glutamine (*Gls2* and *Got1*) after treatment with BI 456906, vehicle, or semaglutide. Significant negative correlation (p < 0.0001, Spearman) was only observed in BI 456906 treatment, but not in semaglutide. R2 values are represented on the plots. ∗p < 0.05, ∗∗p < 0.01, ∗∗∗p < 0.001. DIO, diet-induced obese; TPM, transcripts per million.

GCGR activation alters plasma amino acid concentrations due to its catabolic effects [51]. We therefore measured plasma amino acid concentrations in samples from GLP-1R KO mice (Figure 2C) and the subchronic bodyweight-lowering study (Figure 3). Treatment with BI 456906 in both studies resulted in a dose-dependent reduction of plasma amino acid levels compared with vehicle and semaglutide (Figure 6A–G and S8), suggesting GCGR engagement. This effect was significant for alanine, citrulline, glutamine, ornithine, serine, threonine, and tyrosine. In addition, transcripts for amino acid-metabolizing enzymes were dose-dependently changed with BI 456906 treatment (downregulated *Ddah1*, upregulated *Asns*, *Ass1*, *Gls2*, *Got1*, and *Sds*; Figure 6H–M). We correlated the transcriptional changes for the genes *Sds*, *Gls2*, and *Got1* with the plasma concentrations of serine and glutamine (Figure 6N–P), and for the 93 gene set with the liver cholesterol and triglyceride concentrations (Figure S9). These analyses demonstrated a significant negative correlation, which was not observed for semaglutide, strengthening the conclusion that changes in plasma amino acids are attributed to GCGR agonistic activity of BI 456906 in the liver (Figure 6N–P).

### 3.7 BI 456906 engages the glucagon receptor *in vivo* upon single dosing, increases liver nicotinamide N-methyltransferase mRNA, and reduces plasma serine and glutamine

**Figure 7:**
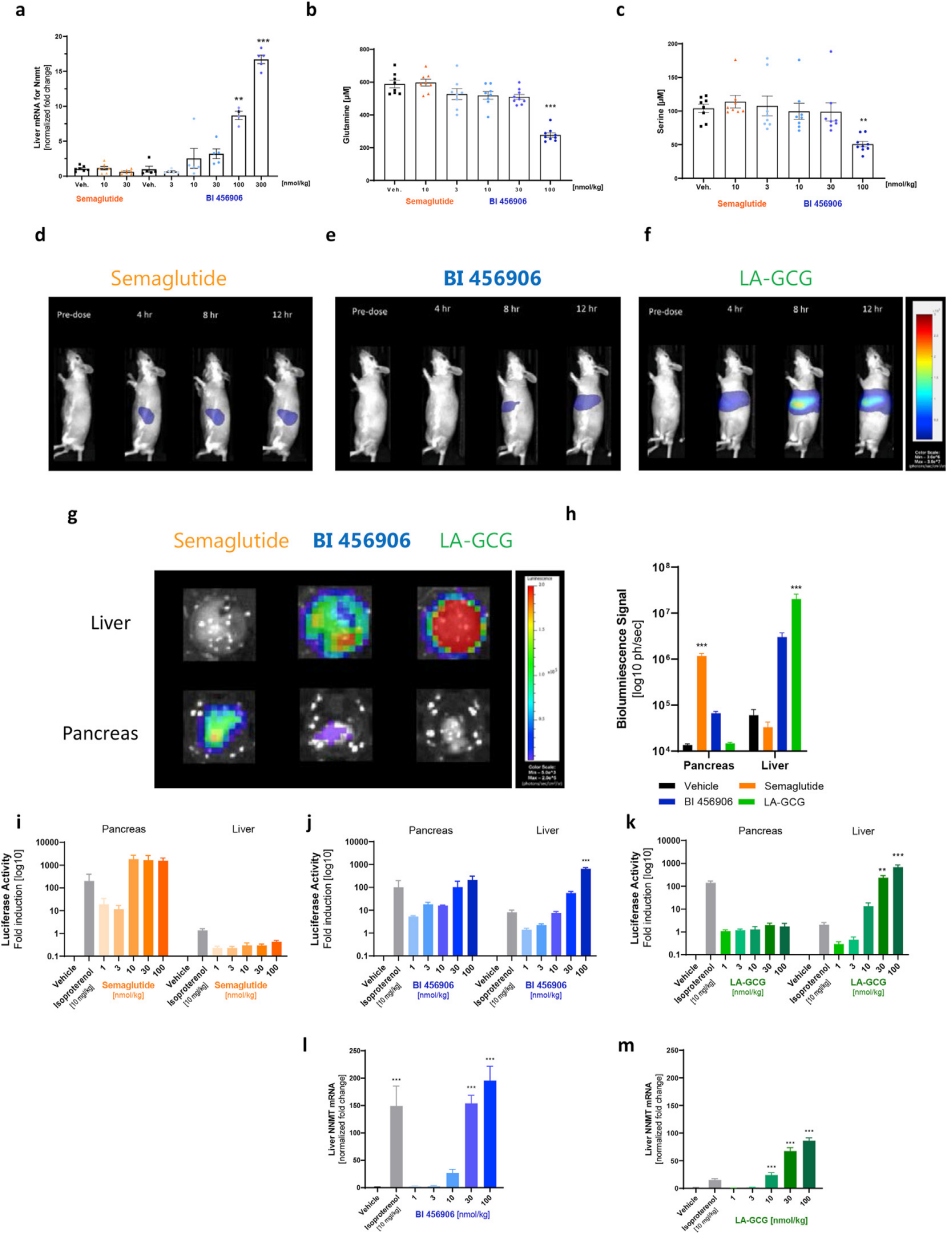
**Effects of BI 456906 on glucagon receptor markers in lean mice and on luciferase activity in target tissues of glucagon-like peptide-1 receptors and glucagon receptors in CRE-Luc mice.** Activation of GCGRs and GLP-1Rs by BI 456906 in lean and in CRE-Luc mice. (a) Liver *Nnmt* mRNA expression after single dosing with vehicle, semaglutide or BI 456906. Plasma levels of (b) glutamine and (c) serine after single dosing with vehicle, semaglutide or BI 456906. Data are shown as mean ± SEM, with individual data shown. (d–m) CRE-Luc mice were dosed with BI 456906, semaglutide and LA-GCG and imaged over 12 h. (d–f) Left lateral imaging of mice pre-dose, 4 h, 8 h, and 12 h after dosing with 100 nmol/kg, (d) semaglutide, (e) BI 456906, or (f) LA-GCG. (g) *Ex vivo* tissue imaging of the liver (top row) and pancreas (bottom row). (h) Bioluminescence signal for *ex vivo* tissue imaging. (i–k) *Ex vivo* determined x-fold induction of luciferase activity in the pancreas and liver after treatment with (i) semaglutide, (j) BI 456906, or (k) LA-GCG versus vehicle and isoproterenol. (l and (m) Liver *Nnmt* mRNA expression in CRE-Luc mice after treatment with (l) BI 456906 or (m) semaglutide versus vehicle and isoproterenol. Data are shown as mean ± SEM. Statistical analysis was done using one-way ANOVA followed by Dunnett’s method for multiple comparisons versus vehicle with significance defined at ∗∗p < 0.01, ∗∗∗p < 0.001. CRE-Luc, cAMP response element-luciferase; GCGR, glucagon receptor; GLP-1R, glucagon-like peptide-1 receptor; Iso, isoproterenol; LA-GCG, long-acting glucagon; NNMT, nicotinamide N-methyltransferase.

Hepatic expression of the *Nnmt* gene was dose-dependently and specifically upregulated by BI 456906, but not semaglutide, implying GCGR agonism in the liver (Figure 5B). Consistent with these findings, single dosing of lean mice with BI 456906, but not semaglutide, dose-dependently increased *Nnmt* mRNA expression in the liver (Figure 7A), reaching significance at 100 and 300 nmol/kg BI 456906 versus vehicle. In addition, plasma amino acids were measured as circulating biomarkers for target engagement of the GCGR; single dosing of mice with BI 456906 significantly reduced plasma glutamine and serine levels compared with control and semaglutide-treated groups at a dose of 10 nmol/kg (Figure 7B and C).

### 3.8 BI 456906 simultaneously engages the GCGR and the GLP-1R in mice upon acute dosing *in vivo*

Simultaneous engagement of the GLP-1R and GCGR *in vivo* by BI 456906 at pharmacologically active doses was compared with semaglutide and LA-GCG, applying the CRE-Luc transgenic mouse model [38], a reporter line that allows the detection of ligand activation of GPCRs to translate *in vitro* to *in vivo* activity. An advantage of this model for the characterization of BI 456906 lies within the use of the same assay principle of CRE-Luc induction as applied to determine *in vitro* receptor potencies (Figure 1C). The suitability of this model was established by single intraperitoneal dosing of the technical control isoproterenol as described [38].

Real-time, *in vivo* imaging was performed up to 12 h. Semaglutide increased luciferase activity in the pancreas, indicating GLP-1R activation (Figure 7D), whereas luciferase activity was observed in the liver for BI 456906 (Figure 7E) and LA-GCG (Figure 7F). To confirm the *in vivo* bioimaging data, individual tissue imaging analysis was performed *ex vivo* to enable respective resolution and quantification at the end of the study (Figure 7G and H). Increased luciferase activity was shown for BI 456906 in the liver and the pancreas, suggesting activation of both the GLP-1R and GCGR, whereas semaglutide showed activity only in the pancreas and the LA-GCG only in the liver, indicating single activation of the GLP-1R and GCGR, respectively (Figure 7G and H).

Liver and pancreas tissues were isolated 12 h after injection. The time point of tissue analysis was established (data not shown), with the maximal response of the luciferase activity approximately 4–5 h after the T_max_ of the respective peptide (Tables S1 and S3). The technical control isoproterenol increased liver and pancreas luciferase activity ∼10- and 1000-fold, respectively (Figure 7I–K). Semaglutide dose-dependently increased luciferase activity in the pancreas (maximal effect at doses ≥10 nmol/kg) with no significant increase in the liver (Figure 7I). By contrast, BI 456906 led to a dose-dependent activation of luciferase activity in both the pancreas and the liver, with maximal responses at 30 and 100 nmol/kg, respectively (Figure 7J). Moreover, LA-GCG dose-dependently increased luciferase activity in the liver only, suggestive of selective engagement of the GCGR (Figure 7K). The dose-dependent engagement of the GCGR by both BI 456906 and LA-GCG was confirmed by an upregulation of *Nnmt* mRNA expression in the livers of CRE-Luc mice (Figure 7L and M), with significant increases seen with 10, 30, and 100 nmol/kg LA-GCG and with 30 and 100 nmol/kg BI 456906. Interestingly, an upregulation of liver *Nnmt* mRNA expression by isoproterenol was noted, consistent with *Nnmt* transcriptional regulation by cAMP (Figure 7L and M).

### 3.9 BI 456906 agonism of the glucagon receptor drives transcriptional changes in mouse hepatocytes that are potentially relevant in humans

**Figure 8:**
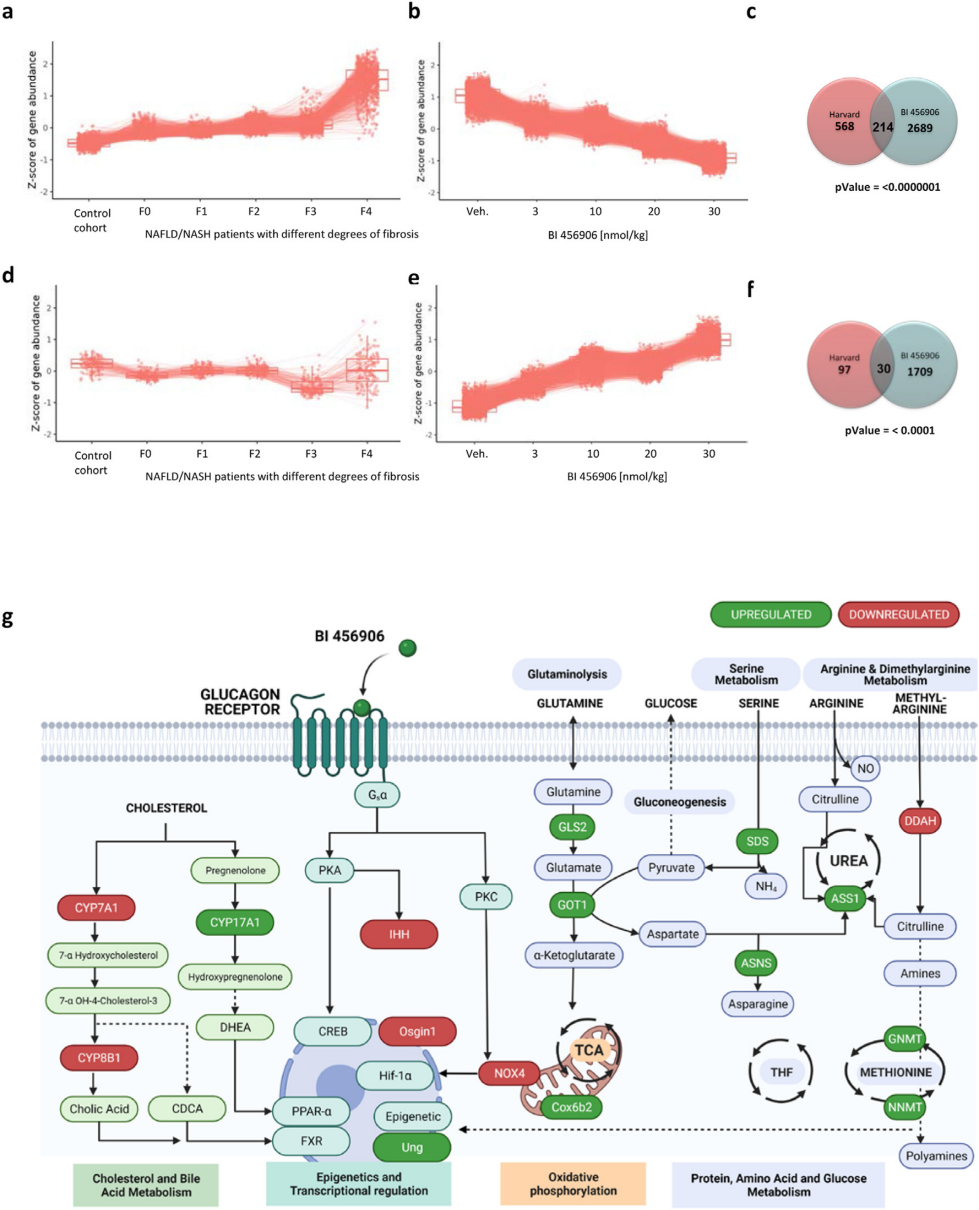
**Gene expression patterns of differentially expressed genes in diet-induced obese mice and humans with non-alcoholic fatty liver disease.** To find BI 456906 dose-dependent genes associated with NAFLD, we compared current study clusters with previously published transcriptomic data on NAFLD by Pantano et al. [46]. (a) Gene clusters that were upregulated over NAFLD fibrosis stages in the Pantano study. (b) Similar gene clusters as in (a) were downregulated with increasing BI 456906 treatment in diet-induced obese mice. (c) The overlap between the clusters was significant (p < 0.0001, hypergeometric test); the Z-score represents the scaled transformation of normalized counts. (d) Gene clusters that were downregulated over NAFLD fibrosis stages in the Pantano study. (e) Similar gene clusters as in (d) were upregulated with increasing BI 456906 treatment. (f) The overlap between the clusters was significant (p < 0.0001, hypergeometric test); the Z-score represents the scaled transformation of normalized counts. (g) Schematic for the working hypothesis of the actions of BI 456906 at GCGRs in the liver, based on transcriptional changes and cluster analyses. GCGR, glucagon receptor; NAFLD, non-alcoholic fatty liver disease; NASH, non-alcoholic steatohepatitis.

To investigate the potential relevance of the transcriptional changes observed in livers from DIO mice treated with BI 456906 in human disease, an unbiased cluster analysis was performed using human liver mRNA sequencing data (Figures 8 and S10) from a cohort of 143 people with obesity (mean BMI 36.7–46.4 kg/m^2^) and concurrent non-alcoholic fatty liver disease or NASH up to fibrosis stage 4 (F0–4). A detailed analysis of this cohort has previously identified multiple clusters of genes that show a strong correlation between gene expression and disease stage [46], shown in Figure 8A and D. The mRNA sequence analysis from livers of mice treated with BI 456906 suggests that gene clusters that are differentially expressed (upregulated, Figure 8A; downregulated, Figure 8D) in the progression of NASH [46] across fibrosis stages in humans exhibited an opposing pattern and were downregulated with increasing doses of BI 456906 (Figure 8B and E), reaching statistical significance (p < 0.001; Figure 8C and F). The same pattern of genes that were downregulated across fibrosis stages in humans (Figure 8D) being upregulated with increasing doses of BI 456906 (Figure 8E) again reached statistical significance (p < 0.001; Figure 8F).

From both the gene expression and cluster analysis (Figure 5, Figure 6 and S10), a working hypothesis for the mechanisms of action of BI 456906 in hepatocytes was derived (Figure 8G), which provides novel insights into GCGR activation by the long-acting dual agonist BI 456906. Our integrated perspective on the GCGR agonistic activity of BI 456906 at therapeutic doses provides evidence for transcriptional changes that go beyond the classical pathways well described for GCGR agonism (cholesterol, bile acid, amino acid, and glucose metabolism) but comprise pathways that are of relevance to transcriptional and epigenetic regulation as well as mitochondrial function (Figure 8G).

## 4 Discussion

BI 456906 is a synthetic peptide designed to simultaneously engage the GCGR and GLP-1R by introducing GLP-1R activity into the glucagon amino acid sequence. To allow dosing of BI 456906 once-weekly as a treatment for people with obesity, the pharmacokinetic profile was optimized by acylation and a specific exchange of amino acid in position 2 to reduce renal clearance and provide resistance to proteolytic cleavage, respectively. Prolonging the *in vivo* half-life of peptides by acylation to promote high-affinity albumin binding is a well-established approach to maximizing the pharmacological potential of peptide hormone mimetics [52]. However, the complexity of peptide lipidation with variations in the lengths of fatty acids, linkers, sites of attachment, and physicochemical properties has been acknowledged in recent years [34,[53], [54], [55], [56]]. Specifically, optimization of these parameters is relevant to their affinities to the target receptors, plasma proteins, and plasma membranes [53,55,57]. All these changes, together with amino acid sequence modifications, influenced the pharmacological profile of the peptide at the target receptors and it was thus important to establish that an appropriate profile had been obtained.

The *in vitro* characterization of BI 456906 included various cellular and functional assays that supported its selection as a balanced dual agonist with an optimized pharmacological profile. BI 456906 species-dependently increased cAMP levels at low potency in hepatocytes endogenously expressing the GCGR, which was not observed for endogenous glucagon. Similar species-dependent potencies in hepatocytes described for cotadutide were thought to be related to differences in GCGR density and thereby the receptor occupancy required to stimulate cAMP [58]. Our data, as well as those of others described here, suggest that receptor pharmacology for acylated peptides requires thorough profiling, as the structural modifications introduced into the peptide sequence are not neutral to the receptor pharmacology, as is seen for receptor kinetics or internalization.

Receptor activation determined by increases in levels of the second messenger cAMP represents a cumulative endpoint that commonly applies a non-selective phosphodiesterase inhibitor, such as 3-isobutyl-1-methylxanthine [59]. Therefore, we complemented our assays with the CRE-Luc format representing a non-cumulative endpoint measure in recombinant cells, as well as functional read-outs in cells of endogenous receptor expression. Importantly, this assay principle allowed us to measure receptor activation in the presence of 100% mouse or human plasma. The potency of BI 456906 for the human GCGR and GLP-1R was differently affected by the presence of mouse or human plasma and was compared with liraglutide, semaglutide, cotadutide, or tirzepatide (Table S4), peptide agonists containing different fatty acid types to extend their *in vivo* half-life [60]. BI 456906 demonstrated a receptor ratio of approximately 1:8 in human plasma, comparable to the once-daily dual agonist cotadutide and different to the 1:3 ratio reported for the recently clinically discontinued dual GCGR/GLP-1R dual agonist NN-1177 in human plasma [34,35]. For the clinical-stage GCGR/GLP-1R agonist cotadutide, the relative receptor ratio *in vitro* in the absence of human plasma was described as 1:5, considered to be optimal for achieving weight loss and glycemic control [58].

Upon single dosing of lean mice with BI 456906 *in vivo*, we demonstrated a dose-dependent engagement of the GLP-1R based on glucose tolerance improvements, acute food intake inhibition, and a reduction in gastric emptying, at a lower potency compared with semaglutide. These measures represent preclinically and clinically well-established physiological responses to GLP-1R agonism and confirm the peripheral and central actions of BI 456906 in engaging the GLP-1R under acute conditions [61].

When administered to DIO mice, 30 nmol/kg BI 456906 demonstrated superior bodyweight-lowering efficacy compared with 20 nmol/kg semaglutide. This superior efficacy correlated with dose-dependent increases in plasma FGF-21 concentrations and dose-dependent reductions in plasma glucagon, cholesterol, and triglycerides, suggesting GCGR engagement. Interestingly, after 4 weeks of subchronic dosing in DIO mice, when food intake was similar to vehicle-treated control animals, semaglutide, but not BI 456906, significantly increased active ghrelin levels. The increase in the orexigenic hormone ghrelin supports an interaction between the ghrelin and GLP-1 system and might be considered as a counter-regulatory response to the anorectic mode of action of selective GLP-1R mono-agonists [62,63].

Various measures were employed that support the conclusion of a simultaneous engagement of the GCGR and GLP-1R by BI 456906 at pharmacological doses [48]. We established an *ex vivo* bioactivity assay as a measure to strengthen the correlation of *in vitro* to *in vivo* activity considering the pharmacokinetic and pharmacodynamic profiles of BI 456906. Importantly, the GCGR agonistic activity of BI 456906 improved, rather than compromised, glycemic control in DIO mice, a profile that appears specific to BI 456906 as this was not observed for other long-acting GCGR/GLP-1R agonists [34]. The increase in energy expenditure by BI 456906 successfully established that the bodyweight lowering in mice was not solely a consequence of food intake reduction. Our findings confirmed the previously established principle of GCGR/GLP-1R dual agonism to provide significantly greater weight loss in preclinical species compared with GLP-1R agonism alone [58,64,65]. Utilizing CRE-Luc reporter mice [38], the simultaneous and dose-dependent engagement of the GCGR and GLP-1R in key target tissues (liver and pancreas, respectively) provided evidence for balanced *in vivo* agonism of BI 456906 at the two receptors. In summary, we applied a thorough *in vitro* and *in vivo* investigation to characterize the relative receptor ratios, which is important given the variable success of other GCGR/GLP-1R dual agonists in clinical development [33].

In the characterization of BI 456906 as a GCGR/GLP-1R dual agonist, we performed a transcriptional analysis of livers from BI 456906- or semaglutide-treated DIO mice. Dose-dependent increases in *Nnmt* mRNA expression in CRE-Luc reporter mice treated with BI 456906, LA-GCG, or isoproterenol, but not semaglutide, confirmed the specific regulation of *Nnmt* by GCGR agonism in hepatocytes. NNMT, which is primarily expressed in liver and adipose tissue, catalyzes the methylation of nicotinamide from S-adenosylmethionine (SAM) to produce S-adenosyl-l-homocysteine (SAH) and methylnicotinamide. Inhibition and siRNA or genetically mediated knockdown of the enzyme has demonstrated considerable metabolic benefits to weight loss, insulin sensitivity, and steatosis in mice [66,67]. However, the functional and causal contribution of NNMT in adipose tissue versus liver under pathophysiological conditions appears incompletely understood. In hepatocytes, *Nnmt* expression was found to correlate with low serum lipids in mice and people with obesity [[68], [69], [70]]. In addition to *Nnmt*, *Gnmt* mRNA was specifically and dose-dependently regulated by BI 456906. GNMT is highly abundant in the liver and is the main enzyme controlling methionine metabolism and the SAM/SAH ratio, using SAM for the synthesis of N-methylglycine. The protective role of GNMT has been well characterized in the context of hepatocellular carcinoma and liver fibrosis in mice and in humans [[71], [72], [73], [74]]. With the identification of *Nnmt* and *Gnmt* as being specifically regulated by BI 456906, agonism of the GCGR may be mechanistically linked to pathways of methionine, polyamine, and nicotinamide adenine dinucleotide metabolism, with potential impacts on cellular energy homeostasis, transcriptional regulation, and epigenetically controlled DNA modifications [70,[75], [76], [77]]. These data warrant further investigations and might provide novel insights into the pharmacology of sustained GCGR activation in the healthy or diseased liver [77,78].

Our approach of correlating plasma amino acid concentrations for serine and glutamine with the regulation of *Sds*, *Gls2*, and *Got1* mRNA suggests persistent engagement of the GCGR by BI 456906 upon subchronic treatment in DIO mice. In agreement with our data, a significant downregulation of *Nnmt*, *Sds*, and *Got1* mRNA in the liver has been described in *Gcg* KO mice [79]. Moreover, changes in plasma amino acid concentrations as biomarkers for GCGR agonistic activity of BI 456906 have been demonstrated to translate clinically [47]. Our exploratory measure of plasma amino acids is limited to a selection of primarily gluconeogenic amino acids; it will be of interest to further extend these initial findings to amino acids such as methionine and branched-chain amino acids, due to their potential relevance for liver function and insulin sensitivity, respectively [80,81]. Furthermore, a more comprehensive analysis of changes in amino acid metabolism by BI 456906 in the context of GCGR agonism and energy expenditure may unravel novel insights into how energy homeostasis adapts to such pharmacological interventions [82]. Interestingly, although changes in expression of mRNA encoding enzymes of gluconeogenesis, such as phosphoenolpyruvate carboxykinase, were expected based on GCGR agonism of BI 456906, no significant changes in the expression of these genes were noted [83].

The transcriptional regulation of genes encoding amino acid- and methionine-metabolizing enzymes appear relevant in the context of preclinical models of NASH, in which fibrosis can be induced with amino acid- or methionine- and choline-deficient (MCD) diets [84]. GCGR/GLP-1R dual agonists consistently showed improvements in NASH and fibrosis in various animal models [48,85,86]; the dual GCGR/GLP-1R agonist G49 demonstrated beneficial effects in MCD diet-induced NASH fibrosis and upon partial hepatectomy in DIO mice, respectively [86]. It is of note that the histopathological improvements observed in the MCD diet NASH model were associated with similar transcriptional changes as described here (e.g. genes of the methionine cycle), suggesting that the identified networks are representative of GCGR agonism rather than a consequence of a particular animal model. Furthermore, the transcriptional changes in genes such as *IHH* [87], *DDAH* [88], *Nox4* [89], and *COX6B2* [90] suggest potential hepatoprotective pathways that comprise anti-fibrotic, anti-inflammatory, and anti-oxidative effects. This is supported by the recent report on the resolution of NASH and hepatic fibrosis by cotadutide and the underlying mechanistic contribution of GCGR agonism [48].

The human relevance of our findings was investigated through applying an unbiased cluster analysis that overlaid the transcriptional networks identified in this study with those identified in people with obesity and NASH at various stages of fibrosis [46]. The enrichment and significance of the overlaying pathways not only included gene clusters with relevance to triglyceride and cholesterol metabolism, but also included clusters representing pathways of extracellular matrix organization and epigenetics. Our data support the hypothesis that the pharmacology of long-acting GCGR agonism exerted by BI 456906 might go beyond the physiology of glucagon as an integrator of glucose, fatty acid, and amino acid metabolism in the liver [91,92]. However, the clinical relevance and therapeutic potential of these mechanisms in promoting hepatocyte and liver integrity [93], function [94], and potentially regeneration remains to be demonstrated [86,95].

In summary, BI 456906 is a potent, appropriately balanced agonist of both the GCGR and the GLP-1R that produces bodyweight reductions through GLP-1R and GCGR dual agonism by the inhibition of food intake, gastric emptying, and increase in energy expenditure, while maintaining normoglycemia in obese, insulin-resistant animals. The preclinical profiling of BI 456906 and its mode of action supports its clinical investigation in Phase II trials in people with overweight/obesity (NCT04667377), diabetes (NCT04153929), and NASH (NCT041771273).

## Data statement

All RNA-Seq raw data used for the present study has been deposited to the Gene Expression Omnibus (GEO): The data from the preclinical animal study is available under accession GSE211105. The data from human NAFLD/NASH patients is available under the accession GSE162694.

## Data Availability

Data will be made available on request.
