# Supplementary material for: BI 456906: Discovery and preclinical pharmacology of a novel GCGR/GLP-1R dual agonist with robust anti-obesity efficacy
Source: Mol Metab. 2022 Nov 7;66:101633. doi: 10.1016/j.molmet.2022.101633 (PMC9679702; doi:10.1016/j.molmet.2022.101633)
Supplement: Multimedia component 1 [file mmc1.pptx]

## Slide 1
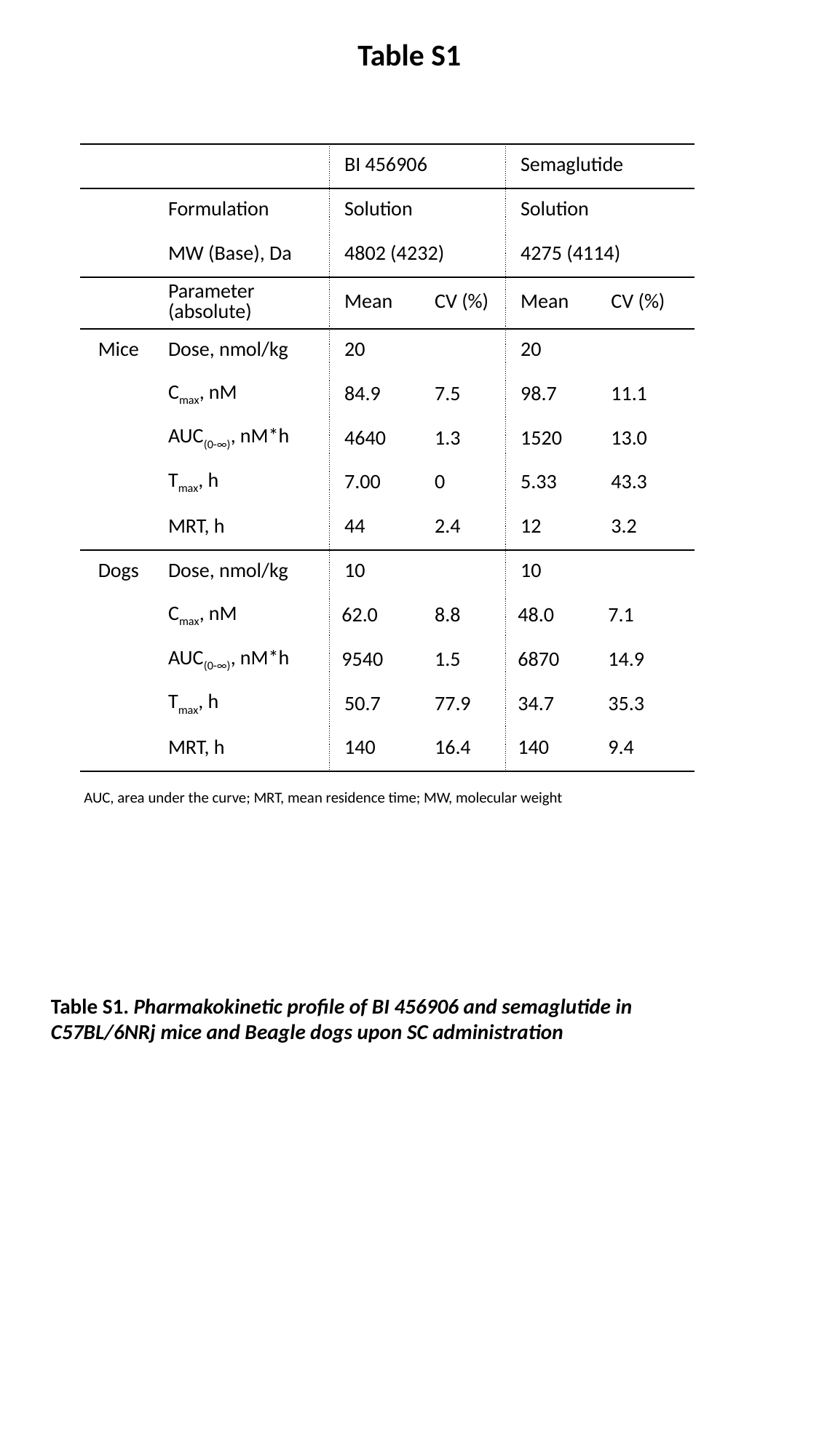

Table S1
| | | BI 456906 | | Semaglutide | |
| --- | --- | --- | --- | --- | --- |
| | Formulation | Solution | | Solution | |
| | MW (Base), Da | 4802 (4232) | | 4275 (4114) | |
| | Parameter (absolute) | Mean | CV (%) | Mean | CV (%) |
| Mice | Dose, nmol/kg | 20 | | 20 | |
| | Cmax, nM | 84.9 | 7.5 | 98.7 | 11.1 |
| | AUC(0-∞), nM\*h | 4640 | 1.3 | 1520 | 13.0 |
| | Tmax, h | 7.00 | 0 | 5.33 | 43.3 |
| | MRT, h | 44 | 2.4 | 12 | 3.2 |
| Dogs | Dose, nmol/kg | 10 | | 10 | |
| | Cmax, nM | 62.0 | 8.8 | 48.0 | 7.1 |
| | AUC(0-∞), nM\*h | 9540 | 1.5 | 6870 | 14.9 |
| | Tmax, h | 50.7 | 77.9 | 34.7 | 35.3 |
| | MRT, h | 140 | 16.4 | 140 | 9.4 |
AUC, area under the curve; MRT, mean residence time; MW, molecular weight
Table S1. Pharmakokinetic profile of BI 456906 and semaglutide in C57BL/6NRj mice and Beagle dogs upon SC administration

## Slide 2
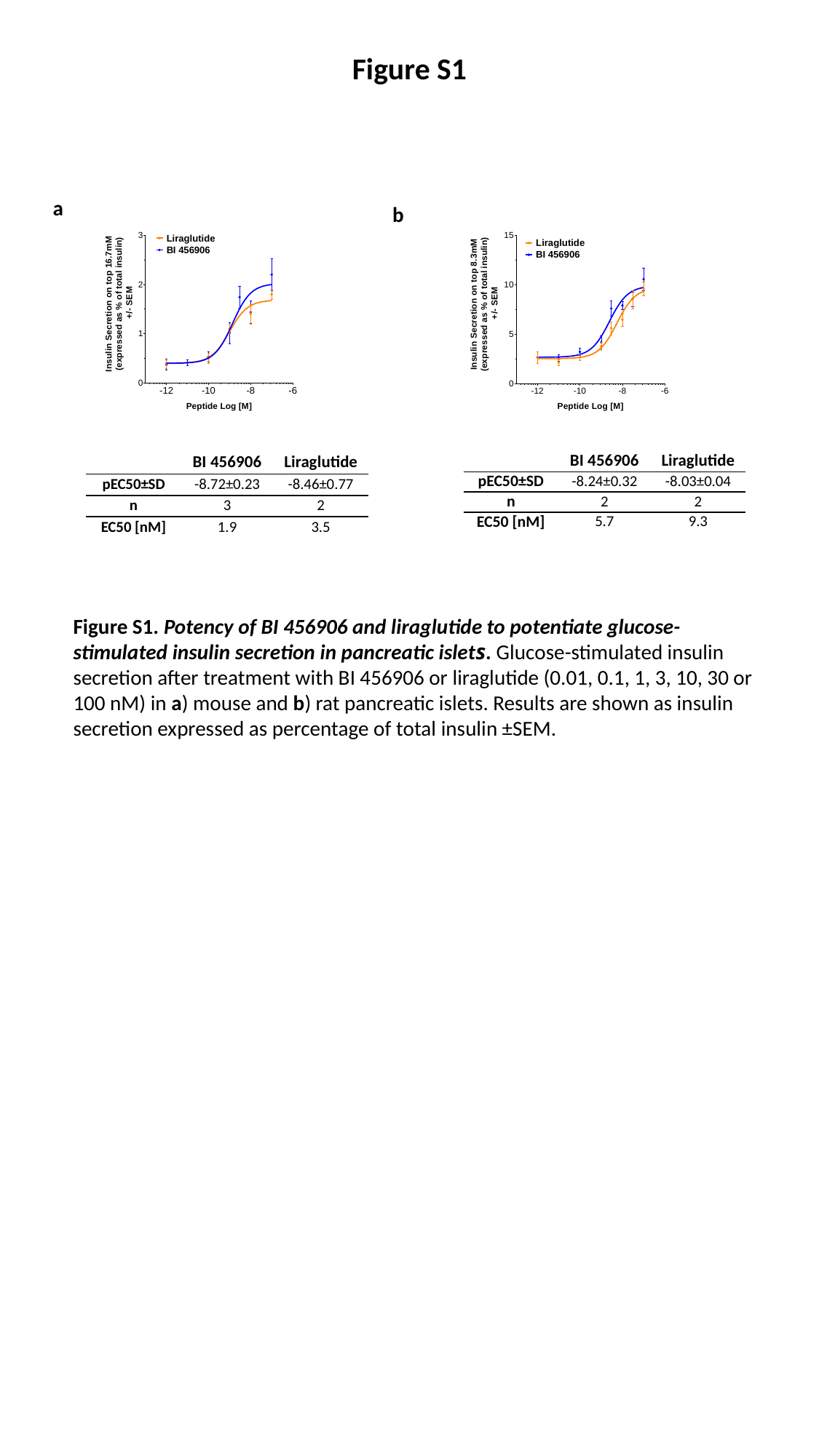

Figure S1
a
b
| | BI 456906 | Liraglutide |
| --- | --- | --- |
| pEC50±SD | -8.72±0.23 | -8.46±0.77 |
| n | 3 | 2 |
| EC50 [nM] | 1.9 | 3.5 |
| | BI 456906 | Liraglutide |
| --- | --- | --- |
| pEC50±SD | -8.24±0.32 | -8.03±0.04 |
| n | 2 | 2 |
| EC50 [nM] | 5.7 | 9.3 |
Figure S1. Potency of BI 456906 and liraglutide to potentiate glucose-stimulated insulin secretion in pancreatic islets. Glucose-stimulated insulin secretion after treatment with BI 456906 or liraglutide (0.01, 0.1, 1, 3, 10, 30 or 100 nM) in a) mouse and b) rat pancreatic islets. Results are shown as insulin secretion expressed as percentage of total insulin ±SEM.

## Slide 3
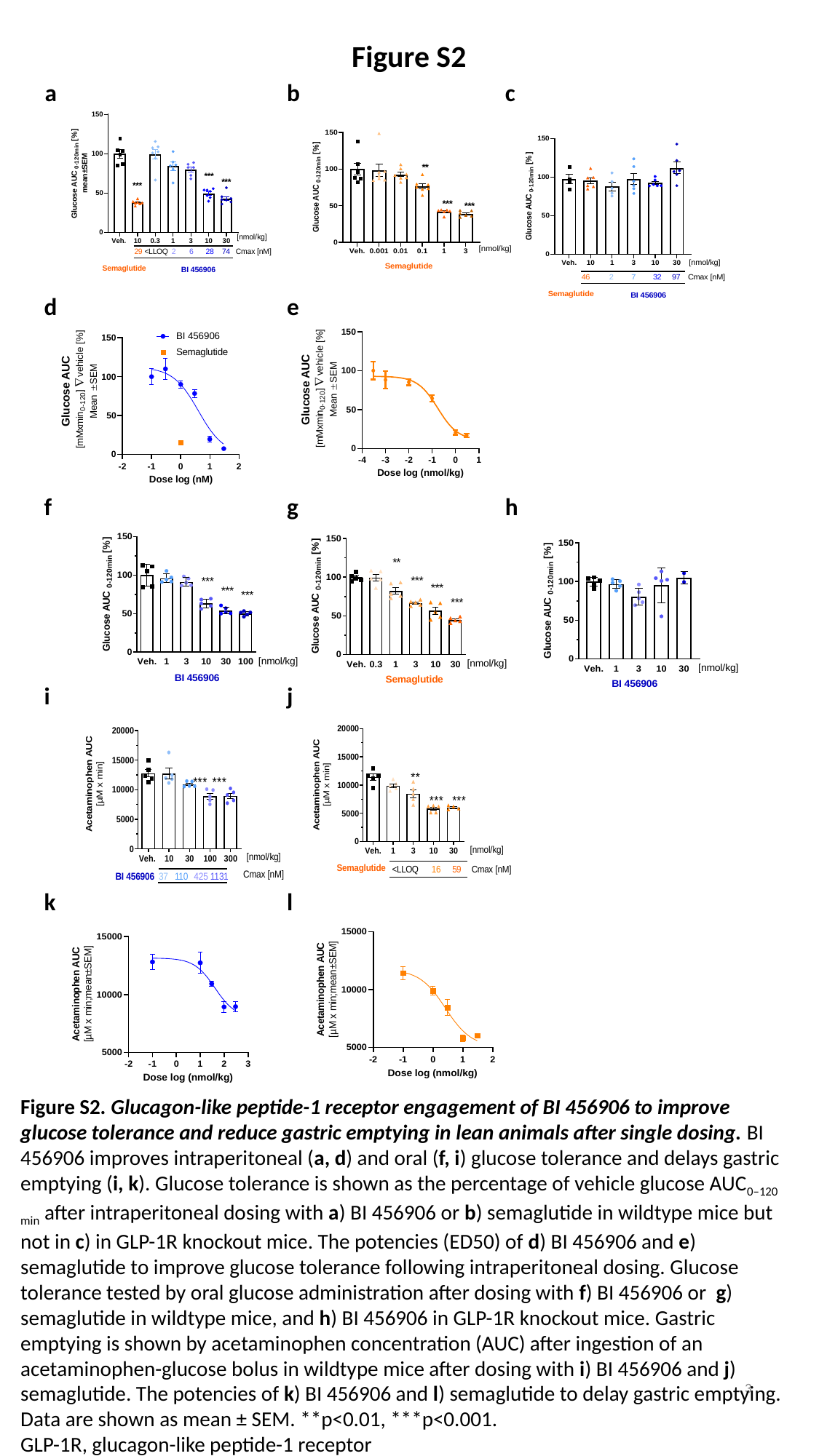

Figure S2
a
d
f
i
k
b
e
g
j
l
c
h
Figure S2. Glucagon-like peptide-1 receptor engagement of BI 456906 to improve glucose tolerance and reduce gastric emptying in lean animals after single dosing. BI 456906 improves intraperitoneal (a, d) and oral (f, i) glucose tolerance and delays gastric emptying (i, k). Glucose tolerance is shown as the percentage of vehicle glucose AUC0–120 min after intraperitoneal dosing with a) BI 456906 or b) semaglutide in wildtype mice but not in c) in GLP-1R knockout mice. The potencies (ED50) of d) BI 456906 and e) semaglutide to improve glucose tolerance following intraperitoneal dosing. Glucose tolerance tested by oral glucose administration after dosing with f) BI 456906 or g) semaglutide in wildtype mice, and h) BI 456906 in GLP-1R knockout mice. Gastric emptying is shown by acetaminophen concentration (AUC) after ingestion of an acetaminophen-glucose bolus in wildtype mice after dosing with i) BI 456906 and j) semaglutide. The potencies of k) BI 456906 and l) semaglutide to delay gastric emptying. Data are shown as mean ± SEM. **p<0.01, ***p<0.001.
GLP-1R, glucagon-like peptide-1 receptor
3

## Slide 4
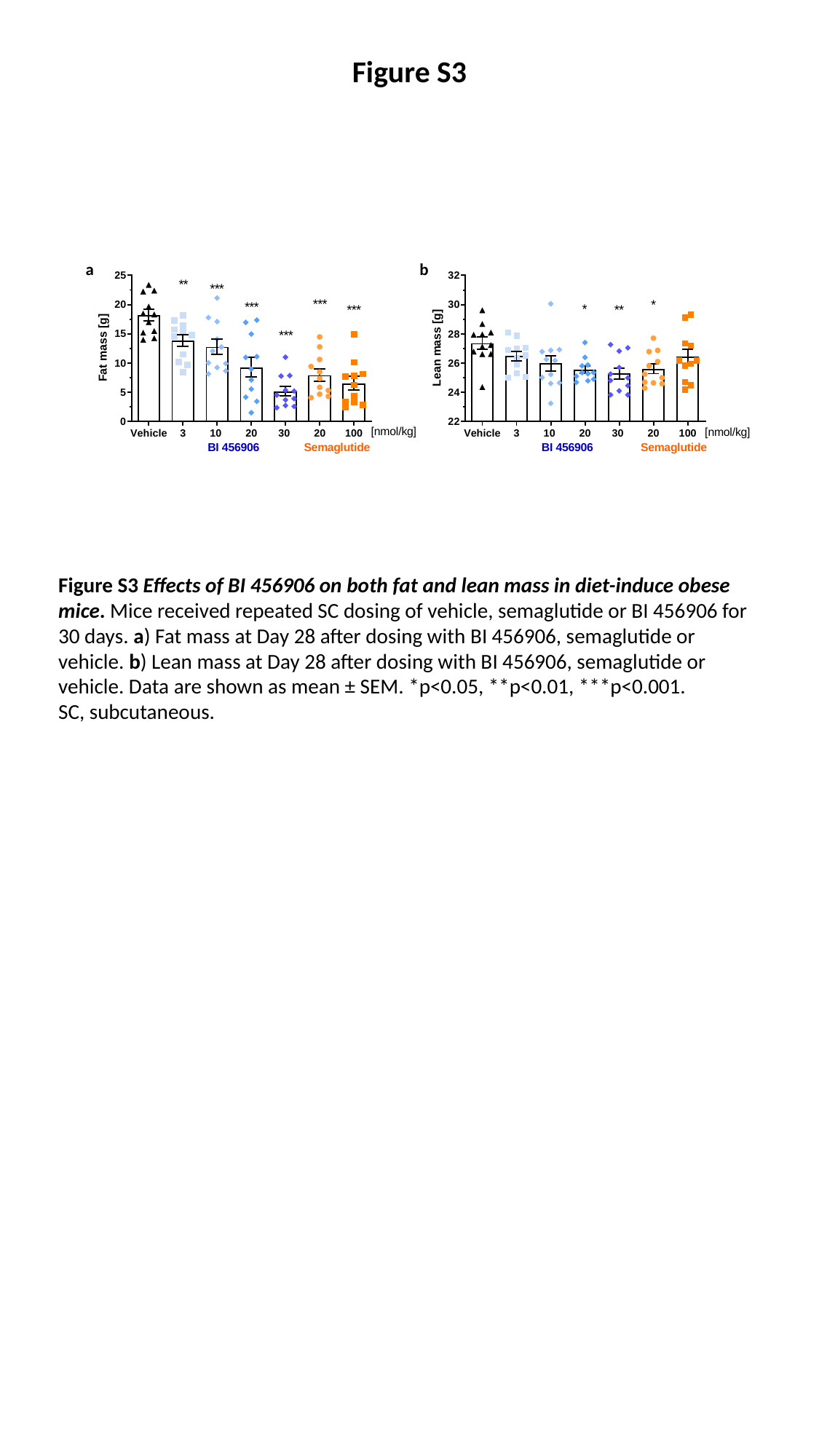

Figure S3
a
b
Figure S3 Effects of BI 456906 on both fat and lean mass in diet-induce obese mice. Mice received repeated SC dosing of vehicle, semaglutide or BI 456906 for 30 days. a) Fat mass at Day 28 after dosing with BI 456906, semaglutide or vehicle. b) Lean mass at Day 28 after dosing with BI 456906, semaglutide or vehicle. Data are shown as mean ± SEM. *p<0.05, **p<0.01, ***p<0.001.
SC, subcutaneous.

## Slide 5
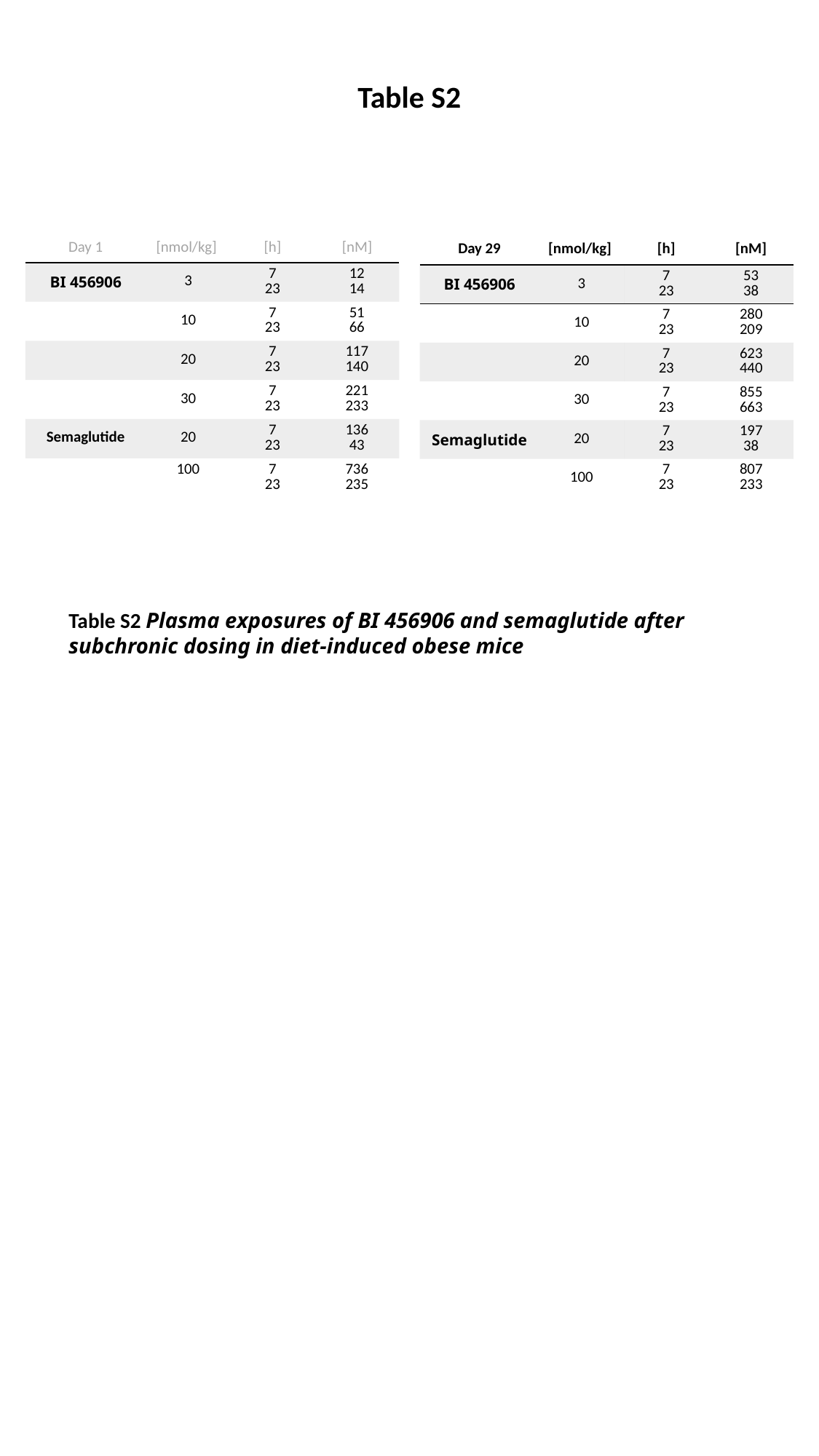

Table S2
| Day 29 | [nmol/kg] | [h] | [nM] |
| --- | --- | --- | --- |
| BI 456906 | 3 | 7 23 | 53 38 |
| | 10 | 7 23 | 280 209 |
| | 20 | 7 23 | 623 440 |
| | 30 | 7 23 | 855 663 |
| Semaglutide | 20 | 7 23 | 197 38 |
| | 100 | 7 23 | 807 233 |
| Day 1 | [nmol/kg] | [h] | [nM] |
| --- | --- | --- | --- |
| BI 456906 | 3 | 7 23 | 12 14 |
| | 10 | 7 23 | 51 66 |
| | 20 | 7 23 | 117 140 |
| | 30 | 7 23 | 221 233 |
| Semaglutide | 20 | 7 23 | 136 43 |
| | 100 | 7 23 | 736 235 |
Table S2 Plasma exposures of BI 456906 and semaglutide after subchronic dosing in diet-induced obese mice

## Slide 6
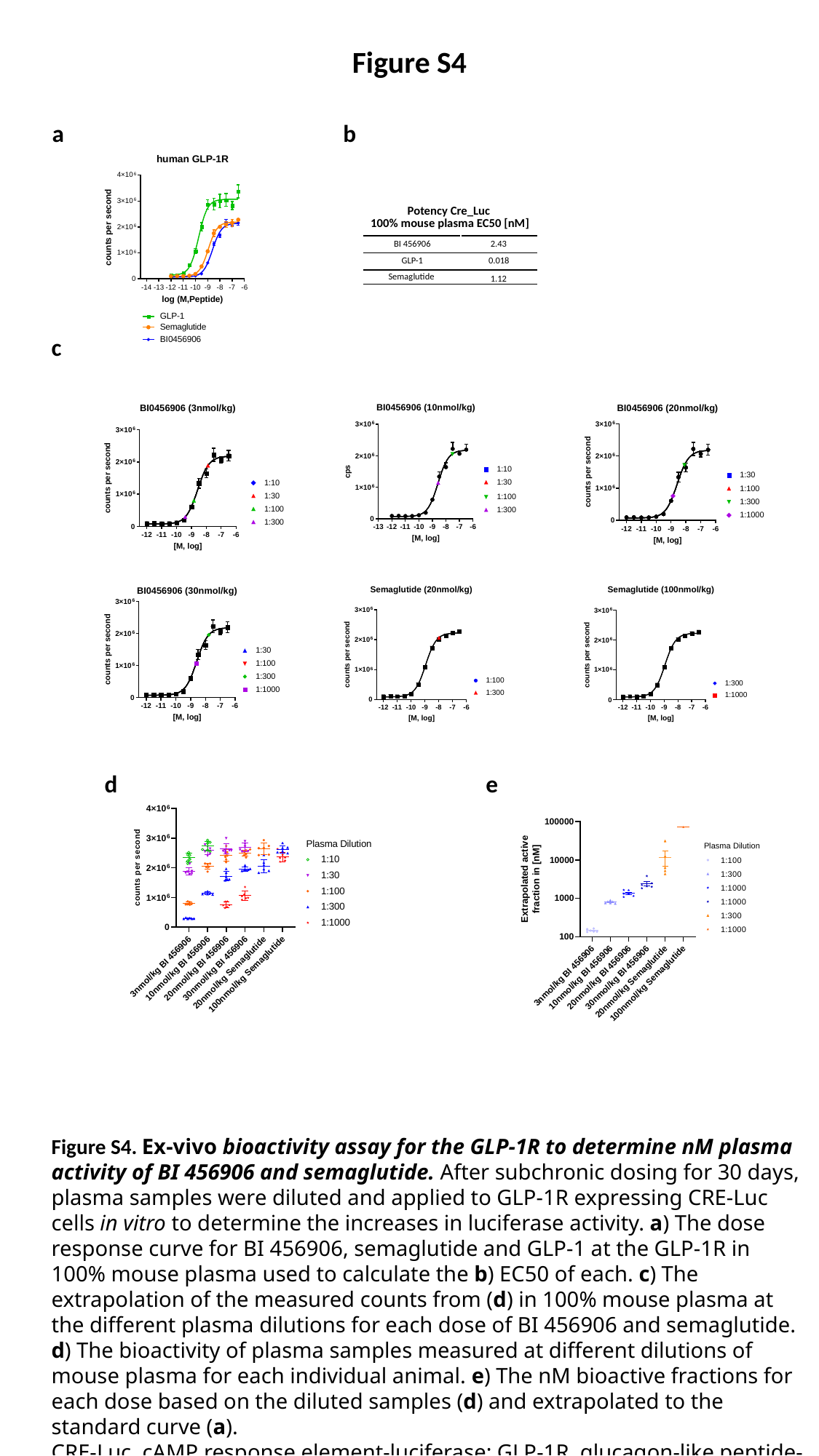

Figure S4
a
b
| Potency Cre\_Luc 100% mouse plasma EC50 [nM] | |
| --- | --- |
| BI 456906 | 2.43 |
| GLP-1 | 0.018 |
| Semaglutide | 1.12 |
c
d
e
Figure S4. Ex-vivo bioactivity assay for the GLP-1R to determine nM plasma activity of BI 456906 and semaglutide. After subchronic dosing for 30 days, plasma samples were diluted and applied to GLP-1R expressing CRE-Luc cells in vitro to determine the increases in luciferase activity. a) The dose response curve for BI 456906, semaglutide and GLP-1 at the GLP-1R in 100% mouse plasma used to calculate the b) EC50 of each. c) The extrapolation of the measured counts from (d) in 100% mouse plasma at the different plasma dilutions for each dose of BI 456906 and semaglutide. d) The bioactivity of plasma samples measured at different dilutions of mouse plasma for each individual animal. e) The nM bioactive fractions for each dose based on the diluted samples (d) and extrapolated to the standard curve (a).
CRE-Luc, cAMP response element-luciferase; GLP-1R, glucagon-like peptide-1 receptor.

## Slide 7
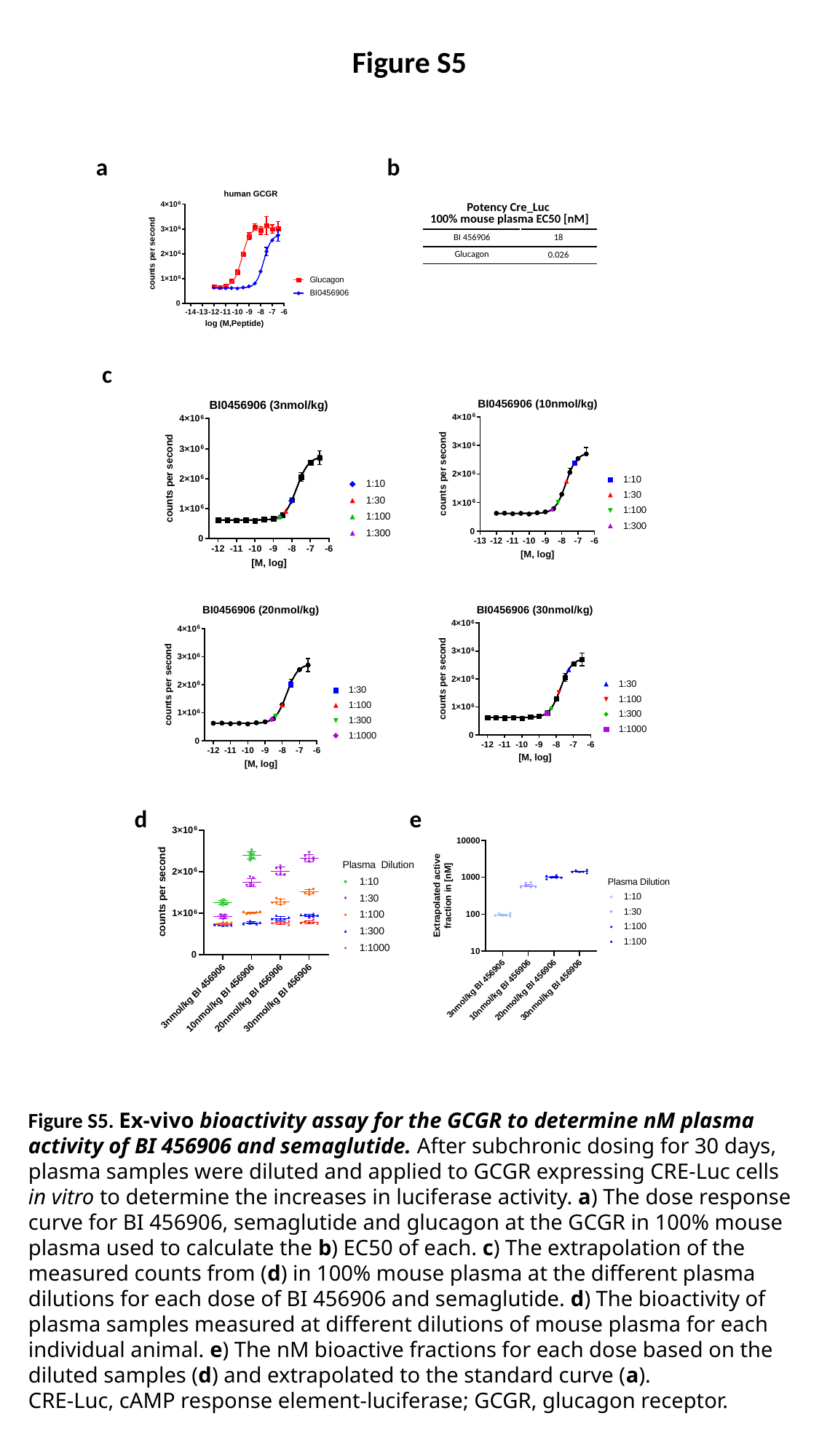

Figure S5
a
b
| Potency Cre\_Luc 100% mouse plasma EC50 [nM] | |
| --- | --- |
| BI 456906 | 18 |
| Glucagon | 0.026 |
c
d
e
Figure S5. Ex-vivo bioactivity assay for the GCGR to determine nM plasma activity of BI 456906 and semaglutide. After subchronic dosing for 30 days, plasma samples were diluted and applied to GCGR expressing CRE-Luc cells in vitro to determine the increases in luciferase activity. a) The dose response curve for BI 456906, semaglutide and glucagon at the GCGR in 100% mouse plasma used to calculate the b) EC50 of each. c) The extrapolation of the measured counts from (d) in 100% mouse plasma at the different plasma dilutions for each dose of BI 456906 and semaglutide. d) The bioactivity of plasma samples measured at different dilutions of mouse plasma for each individual animal. e) The nM bioactive fractions for each dose based on the diluted samples (d) and extrapolated to the standard curve (a).
CRE-Luc, cAMP response element-luciferase; GCGR, glucagon receptor.

## Slide 8
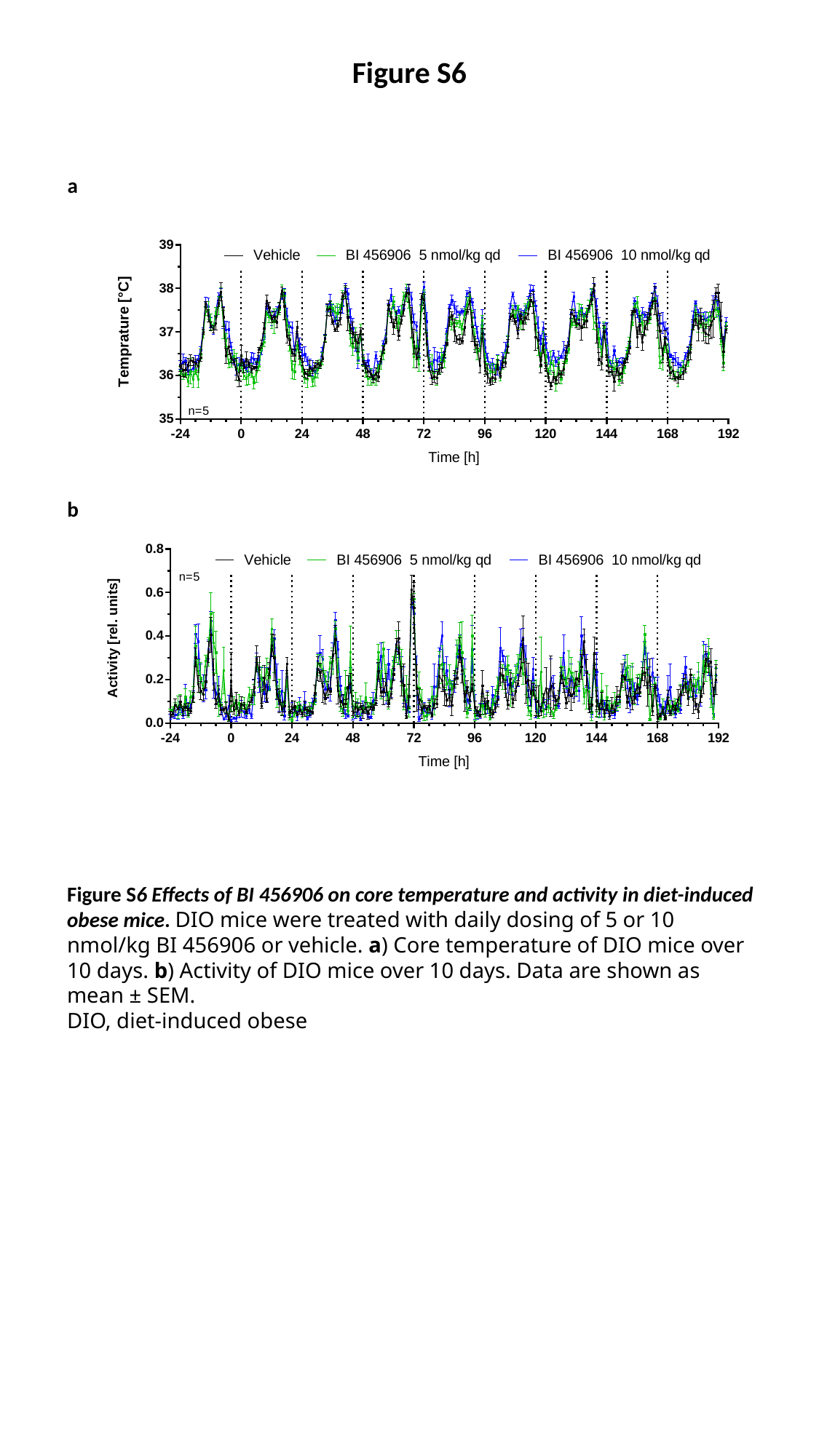

Figure S6
a
b
Activity [rel. units]
Figure S6 Effects of BI 456906 on core temperature and activity in diet-induced obese mice. DIO mice were treated with daily dosing of 5 or 10 nmol/kg BI 456906 or vehicle. a) Core temperature of DIO mice over 10 days. b) Activity of DIO mice over 10 days. Data are shown as mean ± SEM.
DIO, diet-induced obese

## Slide 9
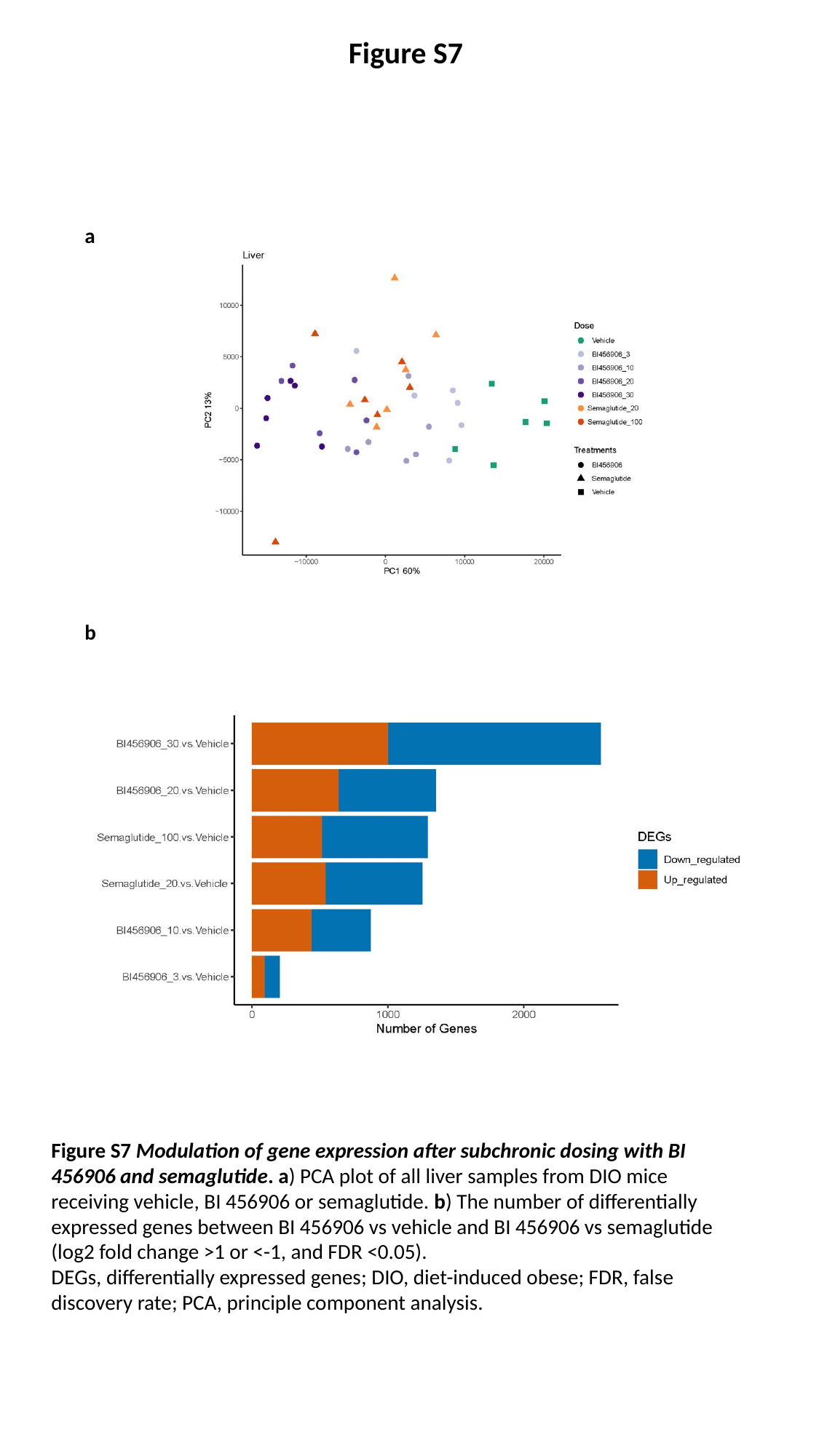

Figure S7
a
b
Figure S7 Modulation of gene expression after subchronic dosing with BI 456906 and semaglutide. a) PCA plot of all liver samples from DIO mice receiving vehicle, BI 456906 or semaglutide. b) The number of differentially expressed genes between BI 456906 vs vehicle and BI 456906 vs semaglutide (log2 fold change >1 or <-1, and FDR <0.05).
DEGs, differentially expressed genes; DIO, diet-induced obese; FDR, false discovery rate; PCA, principle component analysis.

## Slide 10
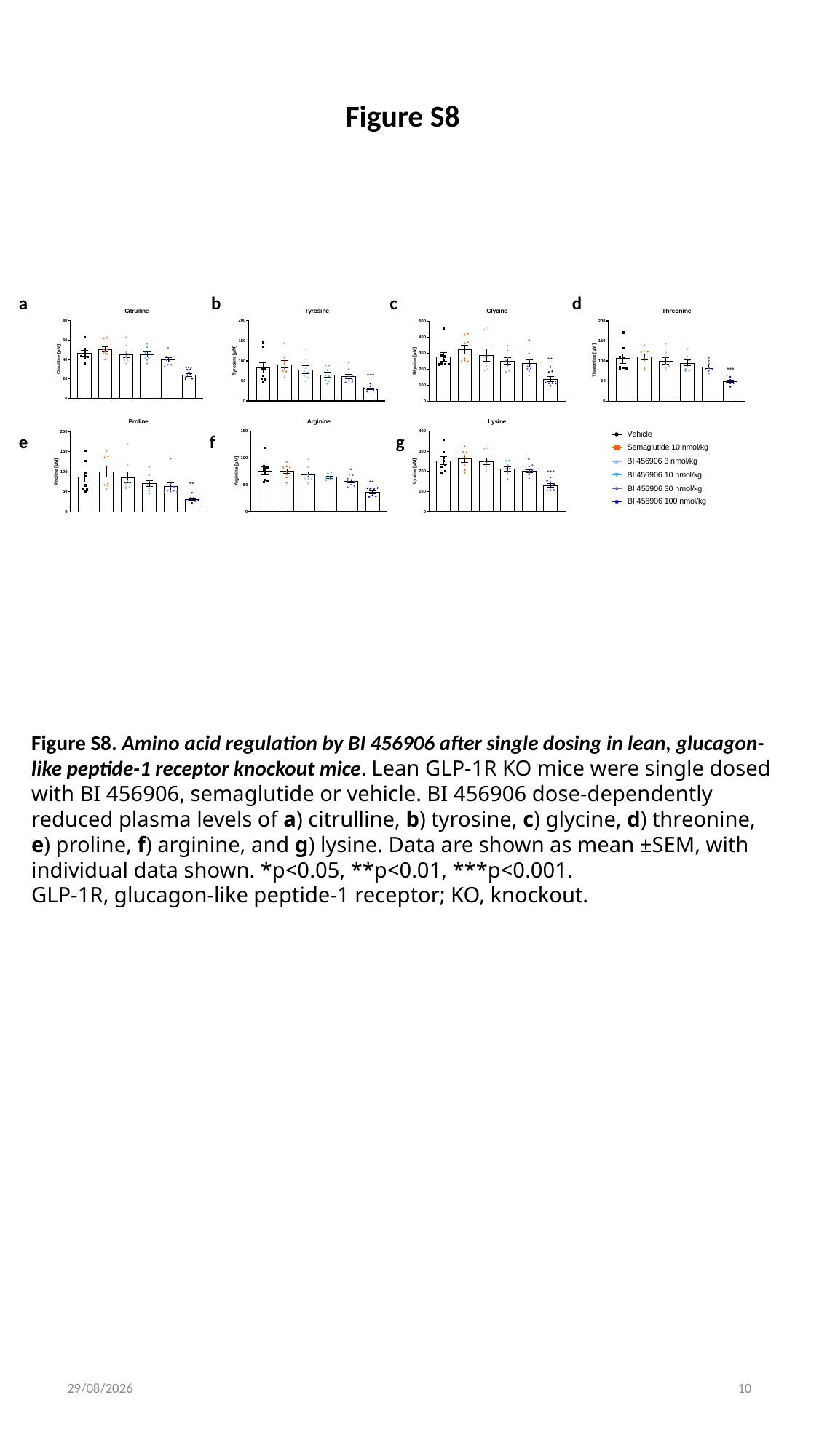

Figure S8
a
b
c
d
e
f
g
Figure S8. Amino acid regulation by BI 456906 after single dosing in lean, glucagon-like peptide-1 receptor knockout mice. Lean GLP-1R KO mice were single dosed with BI 456906, semaglutide or vehicle. BI 456906 dose-dependently reduced plasma levels of a) citrulline, b) tyrosine, c) glycine, d) threonine, e) proline, f) arginine, and g) lysine. Data are shown as mean ±SEM, with individual data shown. *p<0.05, **p<0.01, ***p<0.001.
GLP-1R, glucagon-like peptide-1 receptor; KO, knockout.
17/10/2022
10

## Slide 11
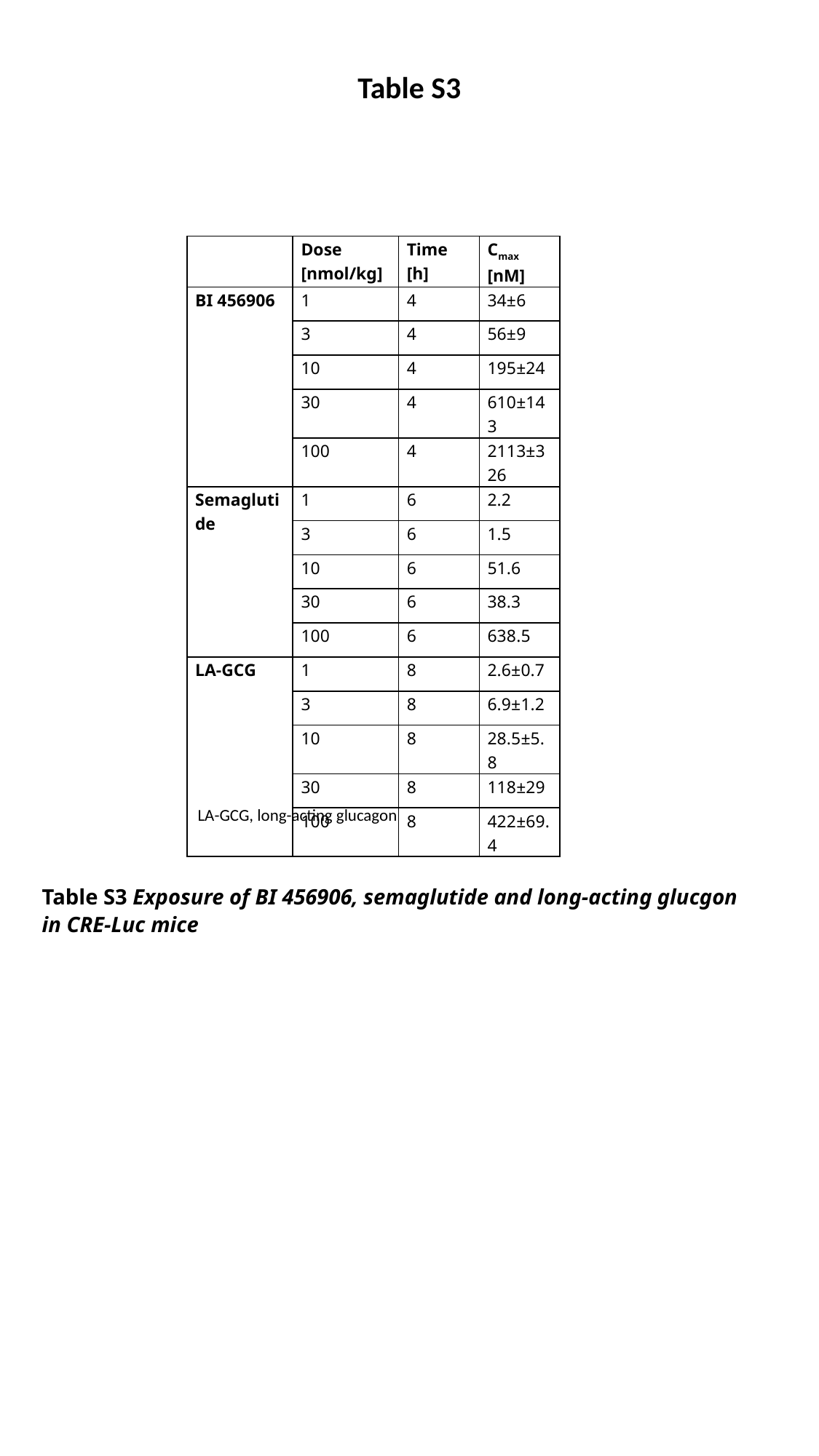

Table S3
| | Dose [nmol/kg] | Time [h] | Cmax [nM] |
| --- | --- | --- | --- |
| BI 456906 | 1 | 4 | 34±6 |
| | 3 | 4 | 56±9 |
| | 10 | 4 | 195±24 |
| | 30 | 4 | 610±143 |
| | 100 | 4 | 2113±326 |
| Semaglutide | 1 | 6 | 2.2 |
| | 3 | 6 | 1.5 |
| | 10 | 6 | 51.6 |
| | 30 | 6 | 38.3 |
| | 100 | 6 | 638.5 |
| LA-GCG | 1 | 8 | 2.6±0.7 |
| | 3 | 8 | 6.9±1.2 |
| | 10 | 8 | 28.5±5.8 |
| | 30 | 8 | 118±29 |
| | 100 | 8 | 422±69.4 |
LA-GCG, long-acting glucagon
Table S3 Exposure of BI 456906, semaglutide and long-acting glucgon in CRE-Luc mice

## Slide 12
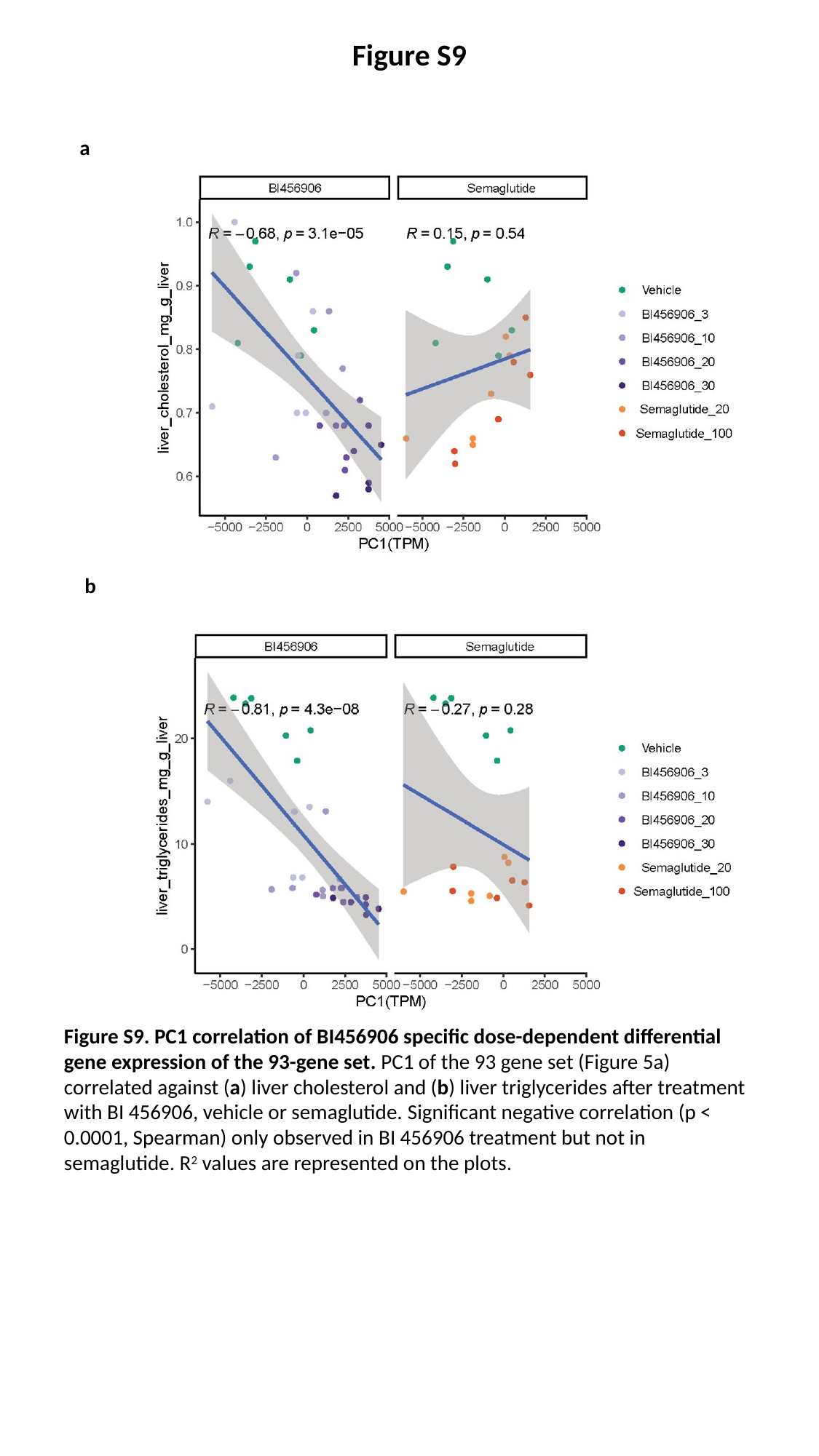

Figure S9
a
b
Figure S9. PC1 correlation of BI456906 specific dose-dependent differential gene expression of the 93-gene set. PC1 of the 93 gene set (Figure 5a) correlated against (a) liver cholesterol and (b) liver triglycerides after treatment with BI 456906, vehicle or semaglutide. Significant negative correlation (p < 0.0001, Spearman) only observed in BI 456906 treatment but not in semaglutide. R2 values are represented on the plots.

## Slide 13
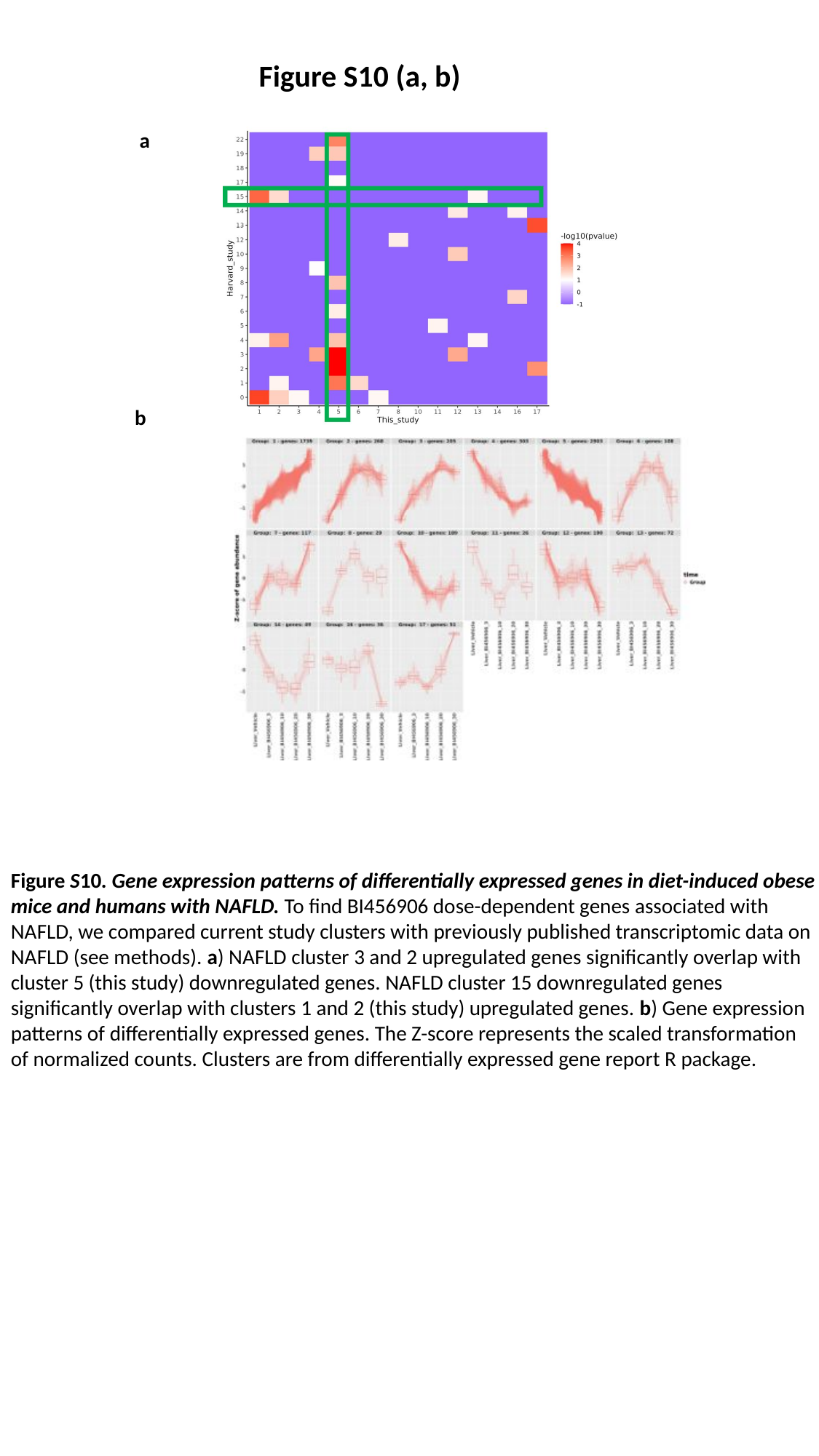

Figure S10 (a, b)
a
b
Figure S10. Gene expression patterns of differentially expressed genes in diet-induced obese mice and humans with NAFLD. To find BI456906 dose-dependent genes associated with NAFLD, we compared current study clusters with previously published transcriptomic data on NAFLD (see methods). a) NAFLD cluster 3 and 2 upregulated genes significantly overlap with cluster 5 (this study) downregulated genes. NAFLD cluster 15 downregulated genes significantly overlap with clusters 1 and 2 (this study) upregulated genes. b) Gene expression patterns of differentially expressed genes. The Z-score represents the scaled transformation of normalized counts. Clusters are from differentially expressed gene report R package.

## Slide 14
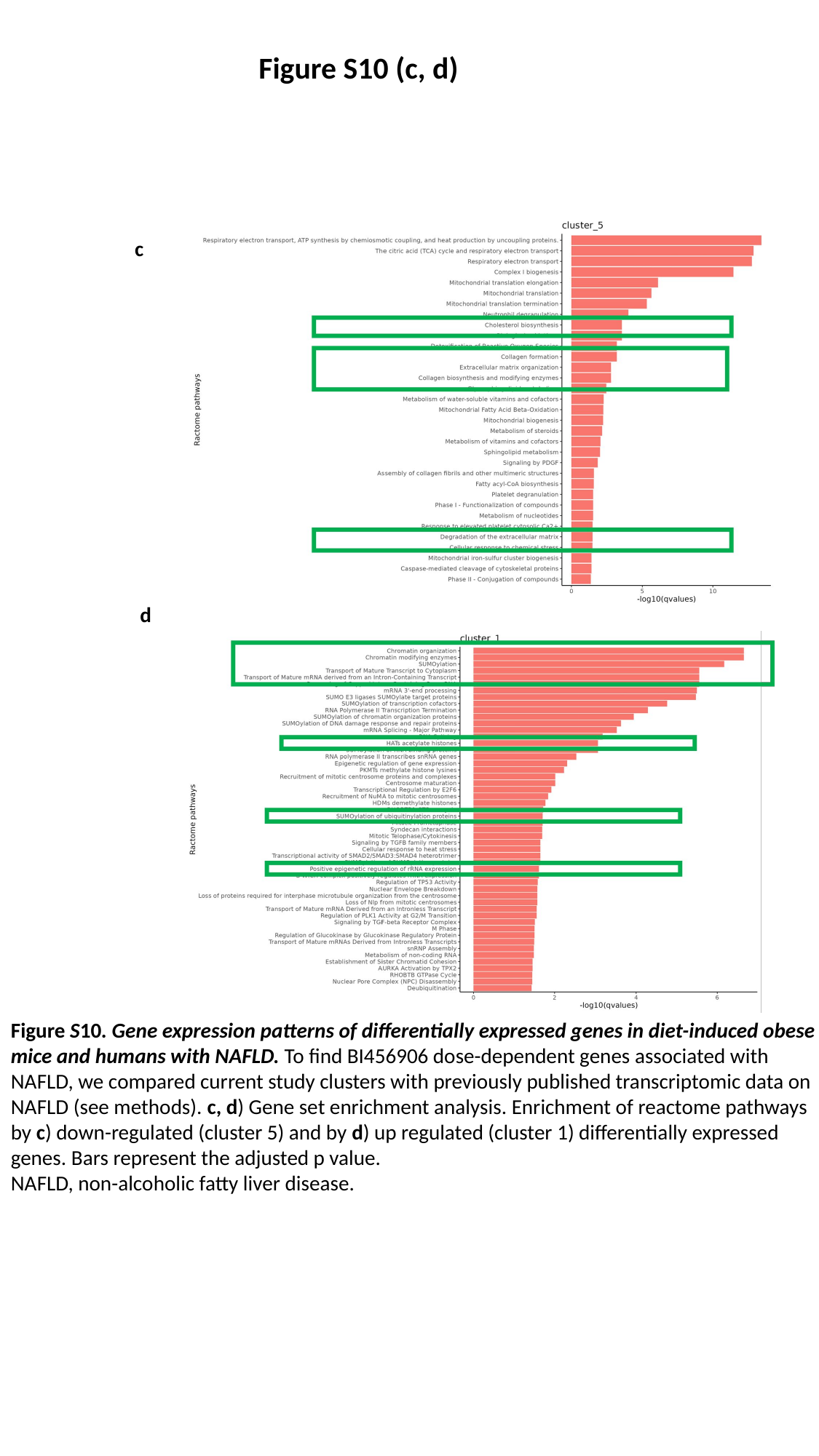

Figure S10 (c, d)
c
d
Figure S10. Gene expression patterns of differentially expressed genes in diet-induced obese mice and humans with NAFLD. To find BI456906 dose-dependent genes associated with NAFLD, we compared current study clusters with previously published transcriptomic data on NAFLD (see methods). c, d) Gene set enrichment analysis. Enrichment of reactome pathways by c) down-regulated (cluster 5) and by d) up regulated (cluster 1) differentially expressed genes. Bars represent the adjusted p value.
NAFLD, non-alcoholic fatty liver disease.

## Slide 15
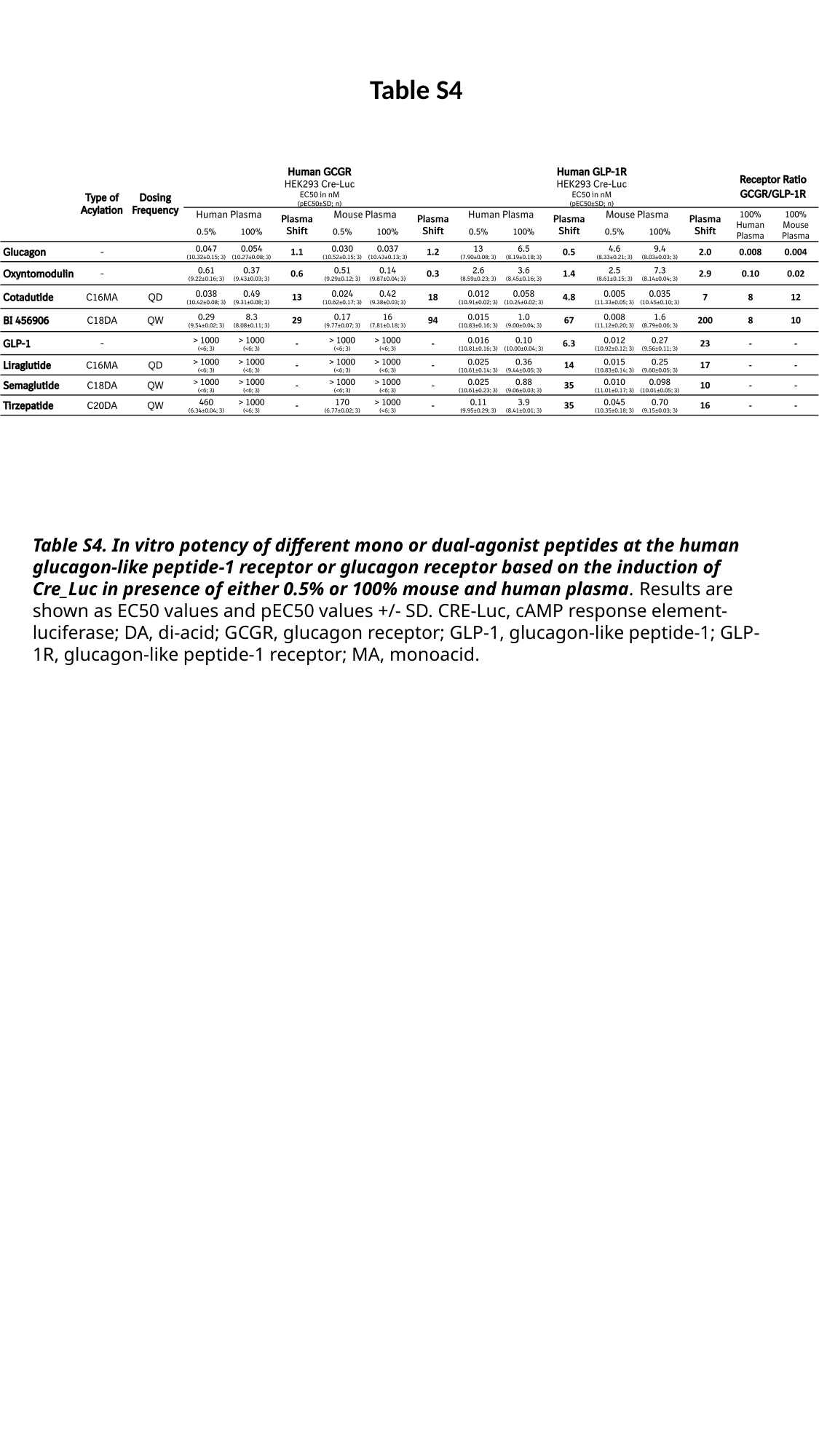

Table S4
Table S4. In vitro potency of different mono or dual-agonist peptides at the human glucagon-like peptide-1 receptor or glucagon receptor based on the induction of Cre_Luc in presence of either 0.5% or 100% mouse and human plasma. Results are shown as EC50 values and pEC50 values +/- SD. CRE-Luc, cAMP response element-luciferase; DA, di-acid; GCGR, glucagon receptor; GLP-1, glucagon-like peptide-1; GLP-1R, glucagon-like peptide-1 receptor; MA, monoacid.
